# Causal role of genetically predicted impairment of branched‐chain amino acid catabolism on insulin secretion and insulin resistance in type 2 diabetes

**DOI:** 10.1111/dom.70565

**Published:** 2026-02-18

**Authors:** Xiangyu Zhou, Jiawen Lu, Zhenqian Wang, Kenneth King‐Yip Cheng, Gloria Hoi‐Yee Li

**Affiliations:** ^1^ Department of Health Technology and Informatics The Hong Kong Polytechnic University Hong Kong SAR China

**Keywords:** branched‐chain amino acids (BCAAs), genome‐wide association study, insulin resistance, insulin secretion, Mendelian randomisation analysis, type 2 diabetes

## Abstract

**Background:**

Elevated branched‐chain amino acids (BCAAs; leucine, valine, isoleucine) are linked to type 2 diabetes (T2D) risk, characterised by defective insulin secretion in pancreatic β‐cell and peripheral insulin resistance. Causative interaction between BCAA metabolism and these two diabetic pathogenesis remains unclear.

**Methods:**

Using publicly available datasets from the European population, we conducted a meta‐analysis of genome‐wide association studies (GWAS), followed by multi‐trait analysis of GWAS (MTAG), to identify genetic loci associated with BCAAs and their catabolites. Two‐sample bidirectional Mendelian Randomisation (MR) examined putative causal associations of genetically determined BCAAs and their catabolites with 10 traits related to insulin and glucose metabolism. Sensitivity analyses evaluated robustness and specificity of observed associations.

**Results:**

MTAG identified 57.14%, 59.09%, and 63.41% novel genetic loci for circulating leucine, valine and isoleucine, respectively. Genetically elevated valine had a significant association with increased insulin fold change during oral glucose challenge test (OGTT) (β [95% CI] = 0.135 [0.045, 0.225]), False discovery rate adjusted *p*‐value (*p*_FDR_ = 0.022), and suggestive association with fasting glucose level (β [95% CI] = 0.031 [0.004, 0.058], inverse‐variance weighted *p*‐value [*p*_IVW_] = 0.025). In the reverse direction, genetically determined homeostasis model assessment of β‐cell (HOMA‐B) exhibited significant inverse associations with BCAAs (Leucine: β [95% CI] = −0.140 [−0.244, −0.036], *p*_FDR_ = 0.034; Valine: β [95% CI] = −0.147 [−0.255, −0.040], *p*_FDR_ = 0.030; Isoleucine: β [95% CI] = −0.149 [−0.248, −0.049], *p*_FDR_ = 0.020). Moreover, β‐hydroxyisovalerate, a leucine‐derived catabolite, was inversely related to 2‐h glucose level after OGTT (β [95% CI] = −0.149 [−0.227, −0.071], *p*_FDR_ = 0.045). In the reverse direction, genetically predicted peak insulin response was suggestively associated with elevated isoleucine catabolite, 2‐hydroxy‐3‐methylvalerate (β [95% CI] = 0.074 [0.018, 0.130], *p*_IVW_ = 9.20 × 10^−3^).

**Conclusions:**

Our genetic analysis indicates BCAA catabolism and insulin secretion/action interact with each other; their aberrance might form a vicious cycle promoting T2D progression.

## 1 INTRODUCTION

Type 2 diabetes (T2D), driven by peripheral insulin resistance (IR) and pancreatic β‐cell dysfunction, afflicts over 10% of the population globally.^1^ In the pre‐diabetic stage, β‐cells secrete more insulin to counteract IR^1^; but overt hyperglycaemia develops when β‐cells become exhausted. Branched‐chain amino acids (BCAAs, leucine, valine, isoleucine), particularly leucine, can directly stimulate insulin secretion in the postprandial state.^2^, ^3^ Their circulating levels are mainly regulated by the coordination among liver, skeletal muscle and adipose tissues. Insulin mediates glucose uptake, and glycogen or triglyceride storage in these metabolic tissues postprandially.^2^, ^4^ However, elevated BCAA levels are positively correlated with glucose dysregulation, IR in obesity, and T2D.^2^ Uncovering the relationship of BCAA metabolism with IR and β‐cell dysfunction in T2D may provide new mechanistic insight and identify potential therapeutic targets.

Not only BCAAs but their catabolites have been shown to contribute to IR and altered insulin secretion. Branched‐chain α‐keto acids (BCKAs), the immediate catabolites of BCAAs, impair insulin signalling and glucose uptake in skeletal muscle cells.^2^, ^5^ Furthermore, 3‐hydroxyisobutyrate (3‐HIB), the valine‐derived catabolite, promotes fatty acid uptake in endothelial cells, leading to ectopic lipid accumulation and IR in skeletal muscle of mice.^6^ Propionyl‐carnitine (C3) and isovaleryl‐carnitine (C5), the distal catabolites of BCAAs, are also implicated in IR.^7^ Under healthy conditions, leucine and its catabolite α‐ketoisocaproate (KIC) act as insulinotropic signals in β‐cell.^8^ The impact of chronic elevation of BCAAs and their catabolites on β‐cell function and insulin sensitivity in humans remain elusive.

Mendelian randomisation (MR) assesses potential causal inference between exposures and outcomes using genetic variants from genome‐wide association studies (GWAS) as instrumental variables (IVs). A unidirectional MR analysis by Lotta et al. identified a significant positive association between genetically determined BCAAs and T2D.^9^ A bidirectional study by Mosley et al. suggested that genetic predisposition to T2D is linked to elevated BCAAs.^10^ Yet, the study lacked evidence supporting the causative effect of BCAAs on T2D, possibly attributed to the limited genetic loci identified for BCAAs and inadequate statistical power. Moreover, these studies largely considered T2D as the only endpoint,^9^, ^10^ leaving uncertainty about which specific T2D‐related pathogenic events drive these associations. Additionally, they did not examine the relationship between BCAA catabolites and T2D^9^, ^10^ (Figure S1, Supporting Information). Here we employed GWAS meta‐analysis and multi‐trait analysis of GWAS (MTAG)^11^ to identify additional loci for BCAAs and their catabolites, followed by testing causal inference across 10 T2D‐related traits using bidirectional MR framework. We demonstrated that genetically elevated BCAAs and their catabolites (i.e., β‐hydroxyisovalerate [HMB]) are significantly associated with impaired glucose clearance, while reduced insulin sensitivity and β‐cell function increase BCAAs. Our findings indicate a vicious cycle between dysregulated BCAA metabolism and impaired insulin/glucose metabolism, which contributes to the progression of T2D.

## 2 METHODS

### 2.1 Study design

**FIGURE 1:**
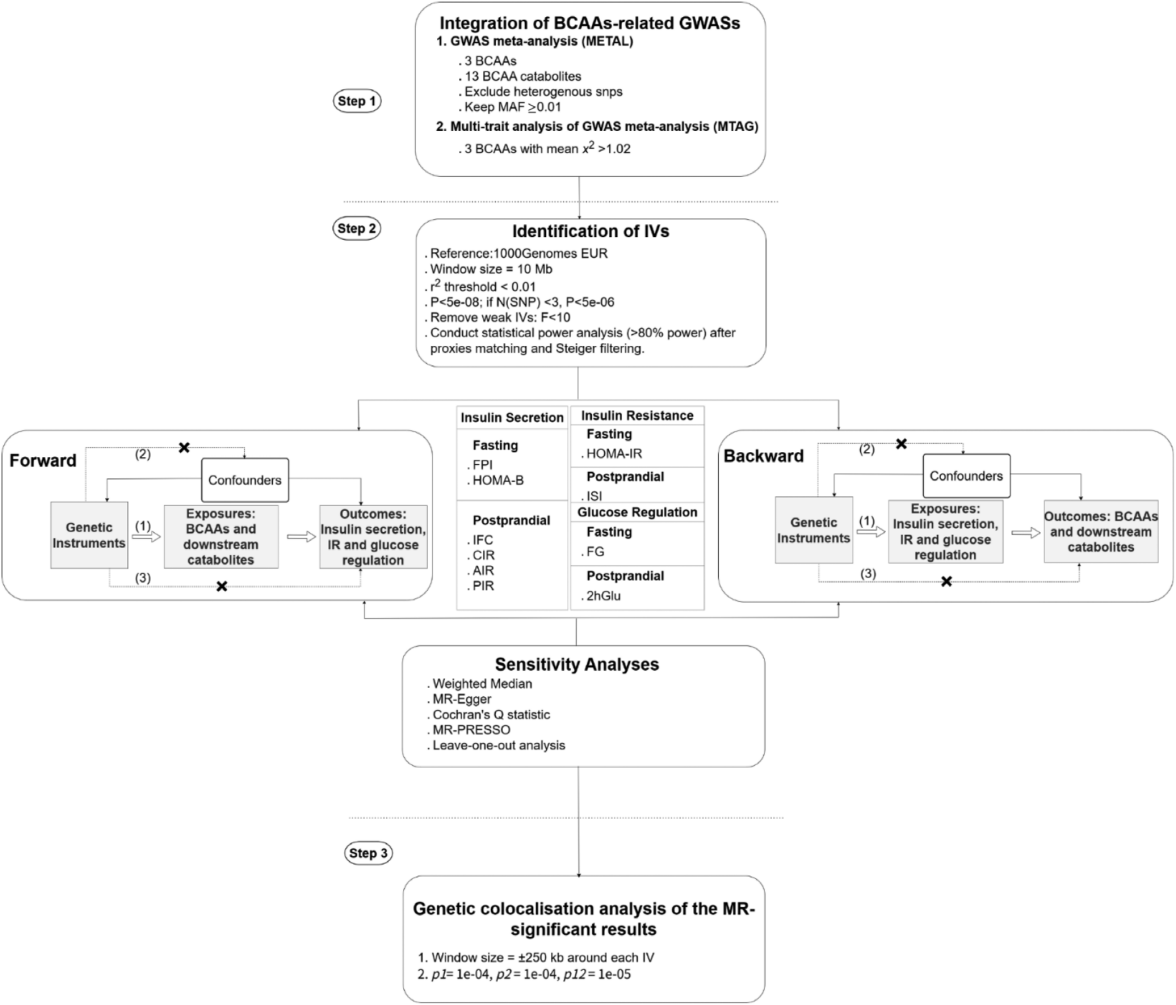
Schematic overview of our study's analytical workflow. (1) Relevance assumptions; (2) independent assumptions; (3) exclusion restriction assumptions; FPI: Fasting proinsulin levels; IFC: Insulin fold change ([Post‐OGTT challenge insulin level]/[fasting insulin level]). 2hGlu, 2 h glucose after Oral Glucose Tolerance Test (OGTT); χ^2^, chi‐square; AIR, acute insulin response; BCAA, branched chain amino acid; CIR, corrected insulin response; FG, fasting glucose levels; GWAS, genome‐wide association studies; IR, insulin resistance; ISI, modified Stumvoll insulin sensitivity index; IVs, instrumental variables; MAF, minor allele frequency; PIR, peak insulin response.

The study design is illustrated in Figure 1. Using publicly available European datasets, we performed GWAS meta‐analysis and MTAG to identify genetic loci for BCAAs and their catabolites, enhancing statistical power (step 1). Genetic variants from step 1 were used as IVs for bidirectional two‐sample MR to evaluate potential causal associations of: (i) BCAAs and BCAA catabolites (exposure) with insulin secretion, IR and glucose regulation (outcome) (forward direction); (ii) insulin secretion, IR and glucose regulation (exposure) with BCAAs and BCAA catabolites (outcome) (backward direction) (step 2). For significant exposure‐outcome associations, we performed colocalisation analysis to assess whether the exposure and outcome shared the same causal genetic variants (step 3).

### 2.2 GWAS meta‐analysis

Inverse‐variance based fixed‐effects meta‐analysis was performed using summary statistics from non‐overlapping GWAS and GWAS meta‐analysis of three BCAAs (Table S1).^12^, ^13^ Genetic loci for 13 BCAA catabolites were identified from either or both the GWAS conducted in the Canadian Longitudinal Study on Aging (CLSA),^14^ and the meta‐analysis of the TwinsUK and Cooperative Health Research in the Region of Augsburg (KORA) datasets^15^ (Table S1). For seven BCAA catabolites with available summary statistics from both studies,^14^, ^15^ inverse‐variance based fixed‐effects meta‐analysis was performed (Table S2). All GWAS meta‐analyses were conducted using METAL,^16^ with effect sizes weighted by the inverse of the squared standard errors (1/SE²) (Data S1).

### 2.3 Multi‐trait analysis of GWAS

MTAG, an extension of inverse‐variance weighted (IVW) meta‐analysis, was used to identify additional genetic loci in the GWAS meta‐analysis of BCAAs with mean chi‐square (*χ* ^2^) ≥1.02.^17^ For BCAA catabolites, summary statistics from single GWAS or our GWAS meta‐analysis were not analysed for MTAG due to the low mean *χ* ^2^ (<1.02), suggesting insufficient polygenic signal and increased risk of false positives^17^ (Data S1). Functional mapping and annotation (FUMA) was employed to define the genomic risk loci identified by GWAS meta‐analysis or MTAG (Data S1).

### 2.4 MR analysis

#### 2.4.1 Data sources

Our GWAS meta‐analysis followed by MTAG (three BCAAs), individual GWAS or our GWAS meta‐analysis (BCAA catabolites) served as data sources for MR analysis in the forward direction. In the reverse direction, IR, insulin secretion and glucose regulation were represented by 10 traits reflecting fasting and postprandial status, with GWAS summary statistics available online (Table S3). Fasting state assesses the baseline and chronic capabilities of β‐cells, whereas postprandial insulin response assessed from oral glucose tolerance test (OGTT) or intravenous glucose tolerance test (IVGTT) reveals acute and dynamic glucose responsiveness.

#### 2.4.2 IV selection

IVs should meet three assumptions: (1) relevance: IVs should be independent of each other and robustly associated with the exposure; (2) independence: IVs are independent of any confounders affecting both exposure and outcome; (3) exclusion restriction: IVs only affect the outcome via the exposure.^18^

Genetic variants having genome‐wide significant association (*p* < 5 × 10^−8^) with the exposure were selected as the IVs. If less than three variants met this threshold, a relaxed threshold of *p* < 5 × 10^−6^ was applied,^19^ provided they had F‐statistics >10 to avoid weak instrument bias. Independent variants were obtained by linkage disequilibrium (LD) clumping, with an *r* ^2^ threshold of 0.01 and a 10 Mb window size. Statistical power of each MR analysis was calculated using an online tool (https://sb452.shinyapps.io/power/ ^20^; Data S1). Adequately powered analyses (≥80%) were prioritised in the main text, while analyses with <80% power were reported in Data S1.

#### 2.4.3 Statistical analysis

We employed IVW method as the primary approach for bidirectional two‐sample MR analysis.^21^ To mitigate potential bias from invalid IVs, we performed several sensitivity analyses, including weighted median,^22^ contamination mixture (ConMix),^23^ MR‐Egger,^24^ and MR Pleiotropy RESidual Sum and Outlier (MR‐PRESSO)^25^ (Data S1).

A potential causal association was deemed statistically significant when the *p*‐value of the primary IVW method (*p*_IVW_) corrected by false discovery rate (FDR; *p*_FDR_) was <0.05. Association with *p*_IVW_ <0.05 but *p*_FDR_ >0.05 was considered suggestively significant. For IVs selected using *p* < 5 × 10^−6^, associations with *p*_FDR_ <0.05 were regarded as suggestively significant. In addition to fulfilling *p*‐value thresholds, the association was only qualified as statistically or suggestively significant if supporting evidence was also obtained from other MR methods: (1) no horizontal pleiotropy (*p*_MR‐Egger_intercept_test_ >0.05, *p*_MR‐PRESSO_Global_test_ >0.05); (2) consistent direction and similar causal estimates across IVW, weighted median, ConMix, MR‐Egger and MR‐PRESSO; (3) statistical significance (*p* < 0.05) in at least one sensitivity analysis. Leave‐one‐out analysis was conducted to assess whether an association was driven by specific IVs.

This study adhered to the Strengthening the Reporting of Observational Studies in Epidemiology using MR (STROBE‐MR) checklist.^26^ All analyses were performed using R (version 4.4.1). Effect estimates were reported as the change in outcome per standard deviation (SD) increase in exposure (β [95% confidence interval, CI]) (Data S1).

### 2.5 Colocalisation analysis

Bayesian colocalisation analysis assessed overlap of genetic signals for exposure and outcome to strengthen causal inference from MR analysis.^27^, ^28^ The window size was ±250 kb centring on each IV,^29^, ^30^ with default priors (*p*1 = 10^−4^, *p*2 = 10^−4^, *p*12 = 10^−5^). The posterior probability for hypothesis 4 (PP.H4) >80% and PP.H4 >50% suggested strong and moderate colocalised associations at a locus, respectively.

## 3 RESULTS

### 3.1 Genetic loci identified for BCAAs and their catabolites

GWAS meta‐analysis followed by MTAG revealed 42, 44 and 41 genomic loci for leucine, valine and isoleucine, respectively (Table 1). Of these, 57.14%, 59.09%, and 63.41% were novel compared to their constituting GWAS datasets^12^, ^13^ and two other published GWAS^31^, ^32^ (Table S4). The increase in mean χ^2^ statistics and higher SNP‐based heritability estimates demonstrated that MTAG enhances association signals and improves genetic contribution estimates for BCAAs (Table 1). While λ_GC_ values were slightly higher in MTAG, LD score regression (LDSC) intercepts were near 1, and negative LDSC ratios confirmed the absence of residual population structure; inflation was due to polygenicity, not confounding. Manhattan (Figure S2) and Quantile‐Quantile (QQ) plots (Figure S3) consistently showed that MTAG identified more genome‐wide significant loci and revealed stronger association signals for BCAAs.

**TABLE 1:** The summary of comparative results of meta‐analysis by METAL and MTAG.

	Leucine		Valine		Isoleucine	
	METAL	MTAG	METAL	MTAG	METAL	MTAG
Genomic risk loci	33	42	37	44	28	41
Lead SNPs	85	104	103	112	61	98
Ind. Sig. SNPs	234	274	294	285	160	249
Mapped genes	27	30	61	75	15	52
Sample size	251 094	263 273	251 068	252 920	251 095	293 746
*χ* ^2^	1.210	1.313	1.244	1.324	1.176	1.302
*h* ^2^(SE)	0.056 (0.0057)	0.065 (0.0065)	0.065 (0.0068)	0.069 (0.0068)	0.049 (0.0040)	0.064 (0.0059)
λ_GC_	1.099	1.194	1.114	1.194	1.093	1.184
LDSC intercept (SE)	0.927 (0.0086)	0.993 (0.0093)	0.925 (0.0090)	0.989 (0.0093)	0.937 (0.0081)	0.986 (0.0091)
LDSC ratio^a^	<0	<0	<0	<0	<0	<0

*Note*: Genomic risk loci: The number of genomic risk loci defined from independent significant SNPs by merging LD blocks if they are less apart than 250 kb. For MTAG, the reported “sample size” is the GWAS‐equivalent sample size, indicating the effective increase in sample size and statistical power from jointly analysing genetically correlated traits. It represents the sample size a standard GWAS would require achieving the same mean association statistic (mean χ^2^) as MTAG. Lead SNPs: The number of lead SNPs reached *p* < 5 × 10^−8^ and were independent each other at *r* ^2^ < 0.1. Ind. Sig. SNPs: The number of independent significant SNPs which reached *p* < 5 × 10^−8^ and were independent at *r* ^2^ < 0.6. Mapped Genes: The number of genes mapped based on positional mapping strategy with a default of 10 kb windows. χ ^2^: chi‐square statistics. *h* ^2^: heritability. λ_GC_: median (χ ^2^)/0.4549. LDSC intercept: LD Score regression intercept. Ratio: (intercept‐1)/(mean (χ ^2^) − 1), it measures the proportion of the LD score regression intercept in the mean χ^2^ that is inflated for reasons other than polygenic heritability.

Abbreviations: METAL, meta‐analysis of GWAS; MTAG, multi‐trait analysis of GWAS meta‐analysis; SE, standard error.

^a^ LDSC ratio <0 usually indicates that the observed inflation has been corrected using genomic control (GC) and is consistent with polygenicity rather than residual population stratification.

Similarly, our GWAS meta‐analysis of seven BCAA catabolites identified 2, 3, 5, 1, 8, 2, 5 genomic loci for KIC, C5, HMB, 3‐methyl‐2‐oxobutyrate (KIV), succinyl‐CoA (C4DC), 3‐methyl‐2‐oxovalerate (KMV), and C3, respectively. There were 100%, 33.33%, 40%, 37.50%, 50% and 20% novel loci identified for KIC, C5, HMB, C4DC, KMV and C3, respectively, compared to the component GWAS^14^, ^15^ (Table S5).

### 3.2 Association of BCAAs with insulin resistance and secretion traits

#### 3.2.1 Forward direction

**FIGURE 2:**
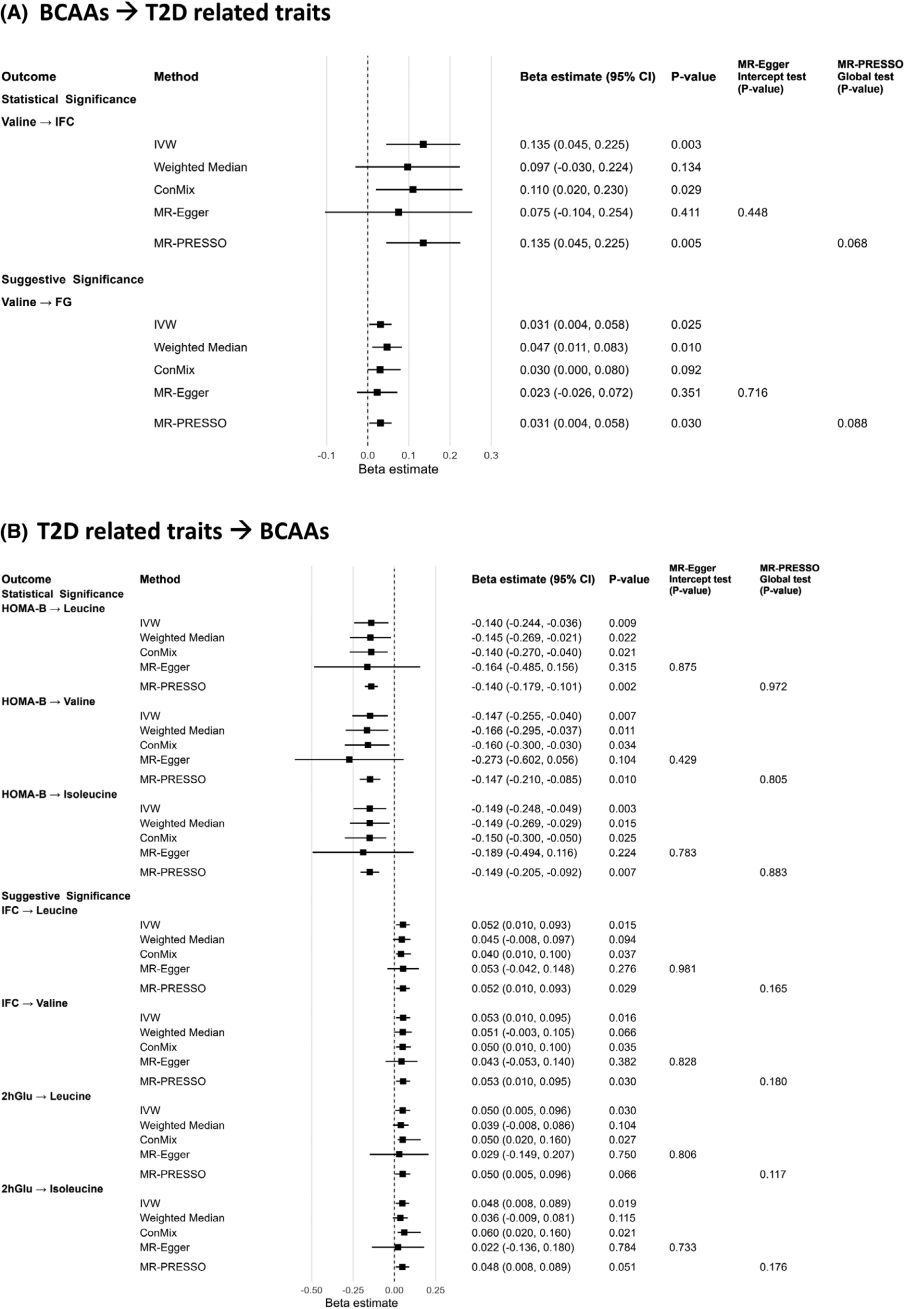
Adequately powered MR analysis between BCAAs and T2D‐related traits. MR analyses with ≥80% power that met statistical or suggestive significance thresholds in the (A) forward and (B) reverse directions are presented. For each exposure–outcome pair, causal effect estimates (β) and 95% confidence intervals (CIs) are shown for the primary inverse variance weighted (IVW) method and sensitivity analyses, including weighted median, contamination mixture (ConMix), MR‐Egger, and MR Pleiotropy RESidual Sum and Outlier (MR‐PRESSO). Horizontal pleiotropy was assessed using the MR‐Egger intercept test and the MR‐PRESSO global test. 2hGlu, 2 h glucose level after an oral glucose challenge test, glucose tolerance; FG, fasting glucose; IFC, insulin fold change.

A total of 51, 54, and 50 SNPs were selected as IVs for leucine, valine, and isoleucine, respectively (Table S6). Statistical power of each MR analysis was presented in Table S7, with adequately powered analysis summarised in Figure 2A and Table S8a. Inadequately powered analysis was summarised in Figure S4A and Table S8b. Genetically elevated valine was significantly associated with increased insulin fold change (IFC) (IVW estimate [β] = 0.135; 95% CI: 0.045, 0.225, *p*_FDR_ = 0.022). Genetically determined valine also had a suggestive positive association with fasting glucose (FG) (β [95% CI] = 0.031 [0.004, 0.058], *p*_IVW_ = 0.025). IVW, weighted median, ConMix, MR‐Egger, and MR‐PRESSO methods yielded consistent direction and similar causal estimates. Neither horizontal pleiotropy nor apparent heterogeneity was observed.

#### 3.2.2 Backward direction

Table S9 lists the statistical power of each backward MR analysis of BCAAs. Adequately powered analyses were presented in Figure 2B and Table S10a, while the remaining analyses were summarised in Figure S4B and Table S10b. Genetically determined HOMA‐B was significantly and inversely associated with leucine (β [95% CI] = −0.140 [−0.244, −0.036], *p*_FDR_ = 0.034), valine (β [95% CI] = −0.147 [−0.255, −0.040], *p*_FDR_ = 0.030) and isoleucine (β [95% CI] = −0.149 [−0.248, −0.049], *p*_FDR_ = 0.020). Using a relaxed IV selection threshold, genetically higher IFC was suggestively and significantly related to increased leucine (β [95% CI] = 0.052 [0.010, 0.093], *p*_IVW_ = 0.015) and valine (β [95% CI] = 0.053 [0.010, 0.095], *p*_FDR_ = 0.049), respectively. No pleiotropy was detected (*p*_MR‐Egger_intercept_test_ and *p*_MR‐PRESSO_Global_test_ >0.05). Additionally, genetically elevated 2hGlu suggestively increased leucine (β [95% CI] = 0.050 [0.005, 0.096], *p*_IVW_ = 0.030) and isoleucine (β [95% CI] = 0.048 [0.008, 0.089], *p*_IVW_ = 0.019).

### 3.3 Association of BCAA catabolites with insulin resistance and secretion traits

#### 3.3.1 Forward direction

**FIGURE 3:**
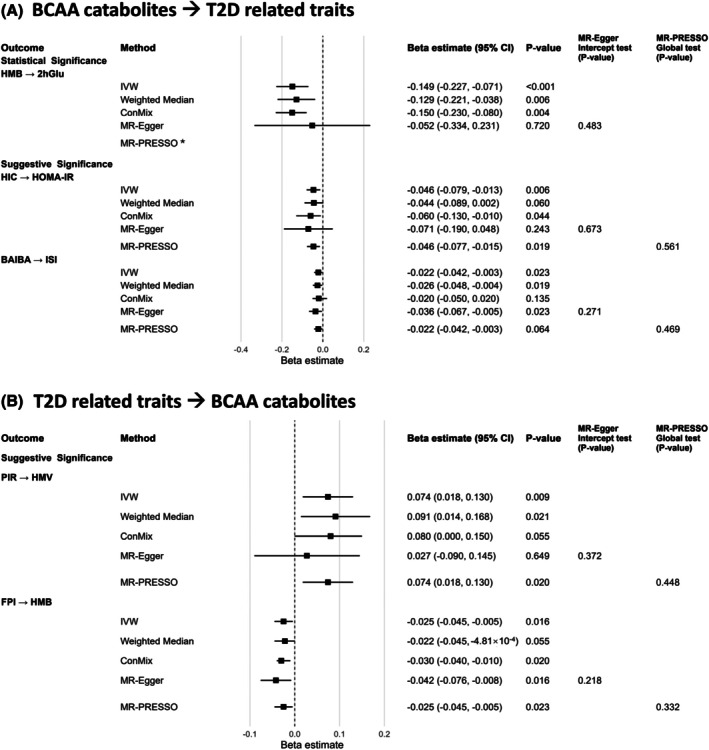
Adequately powered Mendelian randomisation analysis of BCAA catabolites and T2D‐related traits. The forest plots summarise adequately powered MR results assessing causal relationships between genetically predicted BCAA catabolites and T2D‐related traits in the (A) forward and (B) reverse directions. Effect sizes (β) and 95% CIs are shown for inverse variance weighted (IVW) as the primary method, alongside sensitivity analyses using weighted median, ConMix, MR‐Egger, and MR‐PRESSO. Evidence of horizontal pleiotropy was examined via the MR‐Egger intercept test and the MR‐PRESSO global test. *For HMB–2hGlu, MR‐PRESSO could not be conducted (due to 3 IVs only). 2hGlu, 2 h Glucose level after an Oral Glucose Challenge Test, Glucose Tolerance; BAIBA, β‐aminoisobutyric acid; FPI, Fasting Proinsulin; HIC, α‐hydroxyisocaproic acid; HMB, β‐hydroxyisovalerate; HMV, 2‐hydroxy‐3‐methylvalerate; ISI, Modified Stumvoll Insulin Sensitivity Index; PIR, peak insulin response.

For BCAA catabolites, the number of genome‐wide significant (*p* < 5 × 10^−8^) IVs ranged from 4 to 7 for HMB, β‐hydroxyisovaleroylcarnitine (C5‐OH), α‐hydroxyisovalerate (HIV), β‐aminoisobutyric acid (BAIBA), C4DC, and C3. For catabolites with <3 SNPs at *p* < 5 × 10^−8^, we included 7–15 IVs at *p* < 5 × 10^−6^ for KIC, α‐hydroxyisocaproic acid (HIC), C5, KIV, 3‐HIB, KMV, and 2‐hydroxy‐3‐methylvalerate (HMV) (Table S11). Statistical power of each forward MR analysis of BCAA catabolites was reported in Table S12. Analyses with adequate power were summarised in Figure 3A and Table S13a, while remaining analyses were summarised in Figure S5A and Table S13b.

Genetically determined HIC (leucine catabolite) had a suggestively significant inverse relationship with Homeostatic Model Assessment of Insulin Resistance (HOMA‐IR) (β [95% CI] = −0.046 [−0.079, −0.013], *p*_IVW_ = 5.81 × 10^−3^), while HMB showed a statistically significant inverse association with 2hGlu (β [95% CI] = −0.149 [−0.227, −0.071], *p*_FDR_ = 0.045). BAIBA (valine catabolite) showed suggestive inverse relationships with insulin sensitivity index (ISI) (β [95% CI] = −0.022 [−0.042, −3.02 × 10^−3^], *p*_IVW_ = 0.023).

#### 3.3.2 Backward direction

Table S14 reports statistical power for all backward MR analysis of BCAA catabolites. Adequately powered analyses were shown in Figure 3B and Table S15a, and analyses of inadequate power were summarised in Figure S5B and Table S15b. Genetic liability to PIR was suggestively associated with HMV (β [95% CI] = 0.074 [0.018, 0.130], *p*_IVW_ = 9.20 × 10^−3^). Whereas, genetically elevated fasting proinsulin (FPI) had a suggestively inverse association with HMB (β [95% CI] = −0.025 [−0.045, −4.54 × 10^−3^], *p*_IVW_ = 0.016).

### 3.4 Leave‐one‐out analysis

In leave‐one‐out analysis, box plots (Figure S6) indicated that excluding one IV at a time kept most causal estimates tightly clustered around the main estimate without IV exclusion. While excluding certain IVs caused slightly more deviant estimates, there were few outliers and they did not substantially shift overall estimates. Therefore, our MR findings were not excessively driven by any single IV.

### 3.5 Colocalisation of BCAAs and their catabolites with insulin resistance and secretion

**FIGURE 4:**
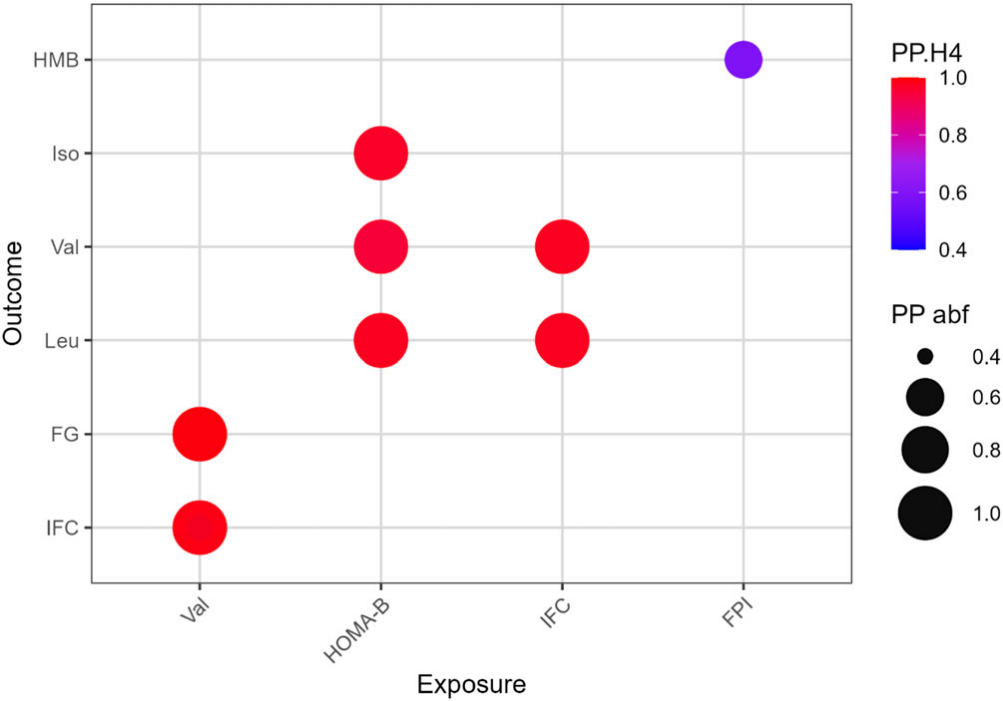
The colocalisation results for the relationship between exposures (on the X‐axis) and outcomes (on the Y‐axis) based on MR results. Each point corresponds to a shared genetic variant linked to both the exposure and outcome, as annotated in Table S16. The size of each point represents the Posterior Probability (PP.abf), with larger points indicating a higher likelihood of association. The colour gradient of the points represents the H4 Posterior Probability (PP.H4), where darker blue indicates weaker evidence of colocalisation and red indicates stronger evidence. posterior probability (PP) for hypothesis 0, 1, 2, 3, 4 (H0, H1, H2, H3, H4). FG, fasting glucose; FPI, fasting proinsulin; HMB, β‐Hydroxyisovalerate; IFC, insulin fold change; Iso, isoleucine; Leu, leucine; Val, valine.

Colocalisation results are summarised in Figure 4 and Table S16. Valine shared rs117643180 with IFC (PP.H4 = 1) and colocalised with FG through rs3817588 (PP.H4 = 0.995). Strong colocalisation was observed between HOMA‐B and each BCAAs at rs11558471 (PP.H4 >0.99). IFC shared rs117643180 with leucine and valine (PP.H4 = 1).

## 4 DISCUSSION

**FIGURE 5:**
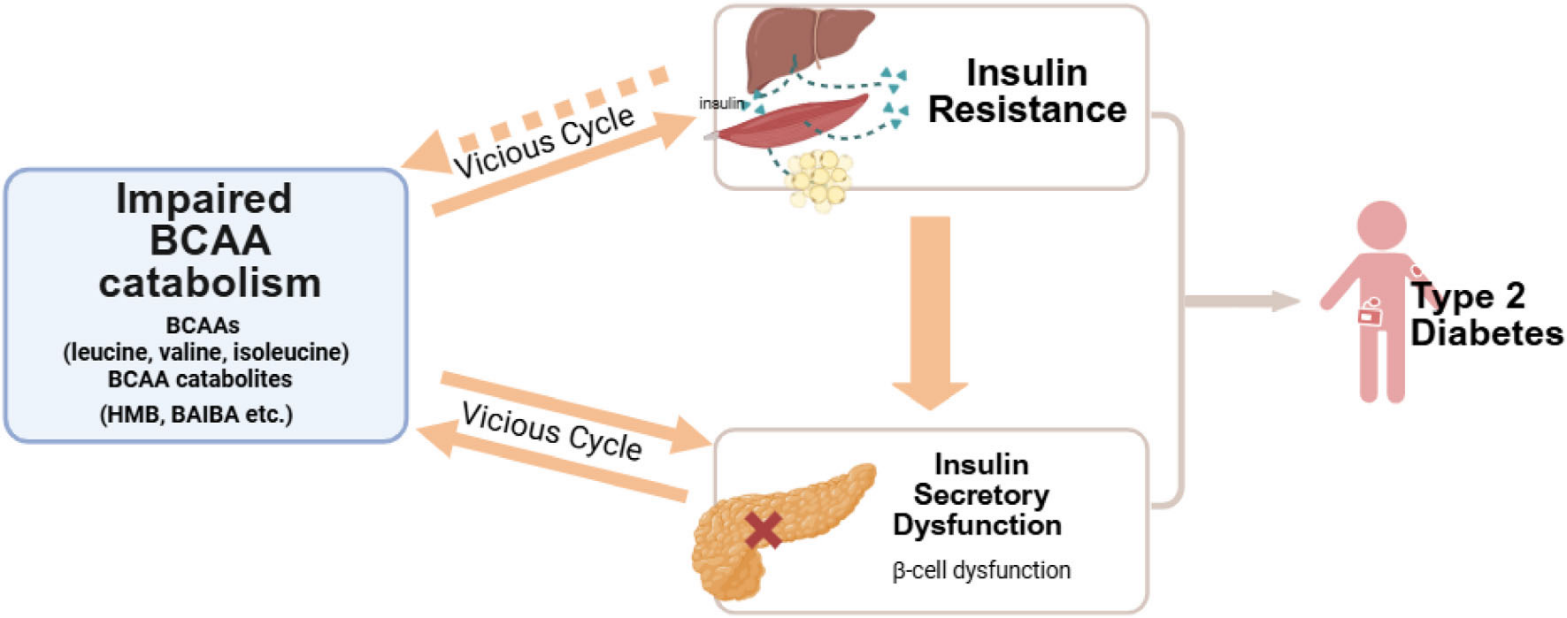
Genetically supported bidirectional relationships between impaired BCAA catabolism and core pathophysiological features of type 2 diabetes. Impaired BCAA catabolism is associated with insulin resistance and β‐cell dysfunction, which in turn may further aggravate BCAA metabolic defects. Dashed arrows indicate exploratory, lower‐powered supplementary MR associations (~75%; ISI–BCAA), whereas solid arrows show associations supported by the main analyses. Together, these findings support a putative vicious cycle that may accelerate T2D progression. BAIBA, β‐aminoisobutyric acid; HMB, β‐hydroxyisovalerate. Created in BioRender.com.

We uncover the bidirectional relationship between genetically determined BCAA metabolism and several key pathogenic features of T2D. While MTAG identified novel genomic loci for circulating BCAAs (42–44 loci, 57%–63% novel), GWAS meta‐analysis revealed 1–8 loci (20%–100% novel) for seven BCAA catabolites. Genetically higher BCAA levels were associated with increased insulin secretion and impaired glucose homeostasis, whereas genetic predisposition to impaired β‐cell function and IR were associated with elevated levels of BCAAs and their catabolites. Based on these findings, we propose the vicious cycle between dysmetabolism of BCAA and insulin/glucose synergistically driving T2D progression (Figure 5).

### 4.1 Statistically significant causal associations identified by MR analysis

Statistically significant associations were defined as primary IVW analyses passing FDR‐correction with supporting sensitivity analyses and no evidence of horizontal pleiotropy. Our robust findings support that proper BCAA catabolism is essential for insulin secretion. Consistently, circulating BCAAs are strongly correlated with postprandial insulin secretion in healthy individuals,^3^ whereas short‐term BCAA restriction reduces postprandial insulin secretion in diabetes.^33^ IFC is also influenced by insulin sensitivity; thus, the actual effect of BCAAs on insulin secretion needs to be confirmed using glucose clamp techniques.

Among the BCAA catabolites, only HMB showed a potential beneficial effect on glucose homeostasis. A previous study showed that HMB supplementation during resistance exercise increased human muscle strength and hypertrophy, which might support insulin sensitivity and glucose disposal in skeletal muscle.^34^ Combination of HMB, lysine and arginine enhanced insulin‐mediated glucose uptake via glucose transporter type‐4 in skeletal muscle of diabetic rat.^35^ Moreover, HMB improved glucose tolerance and increased hepatic insulin‐like growth factor‐1 mRNA expression in mice.^36^ This metabolite also attenuated IR in high‐fructose‐fed rats, potentially through reduced hepatic glucose transporter‐2 expression.^37^ Randomised trials are needed to confirm the beneficial effects of HMB supplementation in people with diabetes.

In the reverse direction, impaired insulin secretion (surrogated by HOMA‐B) may be associated with increased BCAAs. Under obesity and IR, chronic endoplasmic reticulum stress upregulates L‐type amino acid transporter‐1 in β‐cells, thereby increasing BCAA uptake into β‐cells.^38^ The short‐term increase of BCAA may amplify insulin secretion in β‐cells, but its chronic exposure might lead to β‐cell apoptosis and dysfunction.^38^

### 4.2 Suggestive causal associations identified by MR analysis

Several MR analyses reached suggestive significance. We revealed a positive association of genetically predicted valine with increased FG. Consistently, observational studies linked elevated circulating BCAA to IR and glucose dysregulation in children/adolescents^39^ and adults.^40^

Interestingly, our MR analysis revealed a moderate inverse association of BAIBA with ISI. BAIBA, a non‐proteogenic amino acid upregulated by exercise, protects against metabolic diseases in rodents.^41^ Human evidence is inconsistent: one cross‐sectional study demonstrated a positive relationship between circulating BAIBA levels and insulin sensitivity in lean and obese adults,^42^ while another showed no difference in BAIBA between individuals with normal glucose tolerance and pre‐diabetes.^43^ Future longitudinal studies in humans, with adjustment for exercise frequency and intensity, are warranted to clarify the role of BAIBA in insulin sensitivity.^34^, ^35^, ^36^, ^37^, ^39^, ^40^, ^44^

Previous genetic evidence linking glycaemic traits to BCAAs/BCAA catabolites is limited. Our MR study suggests hyperglycaemia may contribute to elevated BCAAs. In contrast, a clamp study showed no effect of acute hyperglycaemia on BCAA levels in rat.^45^ However, MR reflects lifelong systemic effects, including compensatory responses or co‐regulation via insulin. Thus, our findings complement existing mechanistic studies by highlighting the complexity of systemic adaptations to chronic hyperglycaemia beyond direct glucose effects. Despite the limitation of inadequate power (~75%), our MR findings supported the promoting effect of IR (surrogated by reduced ISI) on circulating BCAAs levels (Figure S4B). Our finding aligns with previous MR studies using IR‐associated SNPs^46^ or genetic risk scores based on fasting insulin‐associated variants.^47^ Systemic BCAA levels are affected by dietary intake and BCAA metabolism in the skeletal muscles, white and brown adipose tissues (WAT and BAT), and the livers.^4^ IR and chronic inflammation have been shown to impair BCAA catabolism in the metabolic tissues under diabetic conditions. For example, the BCAA catabolic machinery was downregulated in the metabolic tissues of rodents with obesity. The downregulation was associated with reduced production of the anti‐inflammatory and insulin‐sensitising adipokine adiponectin.^48^ Treatment with the insulin‐sensitising drug rosiglitazone upregulated adiponectin and promoted BCAA catabolism in subcutaneous WAT in mouse model.^49^ In addition, diminished expression of BCAA catabolic genes was associated with reduced expression of PPARγ coactivator‐1α (PGC‐1α) in skeletal muscle of T2D patients. Notably, PGC‐1α was reduced in T2D patients, and its modest increase promoted insulin sensitivity and glucose uptake in skeletal muscle.^50^, ^51^ Skeletal muscle‐specific deletion of PGC‐1α was associated with downregulation of most BCAA catabolic genes in mouse skeletal muscle, whereas PGC‐1α overexpression in primary human myotubes exerted opposite effects.^52^ Taken together, these studies suggest that insulin sensitivity plays a key role in maintaining proper BCAA metabolism.

## 5 STRENGTHS AND LIMITATIONS

Combining large‐scale meta‐analysis, MTAG, and bidirectional MR provides a comprehensive understanding of the entire BCAA metabolic pathway. Consistent sensitivity analyses and strict evaluation thresholds minimise potential biases. However, several limitations remain. First, MTAG can identify additional genetic loci by leveraging genetic correlation across traits but may introduce trait‐specific false positives due to shared genetic signals. False positives in MTAG may be inflated when applied to low‐powered GWAS or to traits with large differences in statistical power. We restricted MTAG to traits with adequate GWAS signal (mean χ^2^ ≥ 1.02) to limit the potential inflation of trait‐specific false positives. Our MTAG analysis was still able to reveal additional loci for BCAAs and increase statistical power for subsequent MR analysis. Second, MR estimates reflect genetic predisposition to elevated BCAAs and BCAA catabolites but cannot directly model/consider gene–environment interactions (e.g., diet, physical activity) or gut microbiota, restricting their true estimation.^53^ Third, despite the use of GWAS meta‐analysis and/or MTAG, the statistical power of several analyses (e.g., HIC with PIR/AIR) remained inadequate, mainly attributed to limited sample size of the outcome GWAS. Fourth, despite the insignificant MR‐Egger intercept and MR‐PRESSO global tests, potential residual pleiotropy necessitates cautious interpretation. Fifth, our analysis was restricted to European‐ancestry populations, limiting the generalisability of findings to other ancestries where allele frequencies, LD patterns, physiology (e.g., muscle mass), lifestyle factors (e.g., diet) may alter BCAA‐T2D relationships. Finally, although the relatively small effect sizes observed for individual metabolites may not be clinically significant, the combination of multiple metabolites for risk profiling may have meaningful clinical utility, potentially yielding population‐level impact. Future studies with increased sample size from diverse populations are warranted to validate our novel genetic loci, enhance statistical power, assess generalisability of our findings across ancestries, and explore potential population‐specific effects.

## 6 CONCLUSION, CLINICAL IMPLICATIONS AND FUTURE DIRECTION

Our study suggests that dysregulated BCAA metabolism, IR, and β‐cell dysfunction are closely interconnected in T2D. These pathogenic factors synergise to create the viscous cycle that initiates and accelerates T2D. BCAAs and their catabolites may serve not only as biomarkers but also as potential therapeutic targets for T2D. Indeed, emerging evidence suggests that reducing circulating BCAA levels exerts beneficial effects on glucose metabolism and insulin sensitivity in humans or rodents with diabetes. For example, sodium phenylbutyrate (NaPB), an FDA approved drug for urea disorder, has been repurposed to improve peripheral insulin sensitivity in T2D patients.^54^ This drug inhibits branched‐chain α‐ketoacid dehydrogenase kinase (BCKDK) activity, thereby promoting BCAA catabolism and reducing their circulating level. A recent clinical trial also showed that a 4‐week dietary restriction of BCAA improves postprandial glucose sensitivity, mitochondrial function in WAT and alters gut microbiome composition in T2D patients.^33^ Using a more specific and potent compound promoting BCAA catabolism (such as PF‐07208254)^55^ and monitoring long‐term effects of low BCAA diet on T2D and its metabolic traits warrant further investigation.

Beyond glycaemic regulation, BCAA catabolism is essential for heart function and T2D increases the risk of heart diseases. A lower ratio of BCAA/aromatic amino acids and leucine/phenylalanine has been associated with worse prognosis and a higher risk of cardiac events in humans with heart failure.^56^, ^57^, ^58^ Additionally, a reduced BCAA‐to‐total amino‐acid ratio has been linked to adverse outcomes in patients with non‐ischemic dilated cardiomyopathy.^59^ It is worth exploring the causal relationship between BCAA metabolism and heart functions in humans using MR approaches in future studies.

To conclude, combining BCAA catabolic and insulin‐sensitising therapies may offer a promising and better strategy for T2D management.

## AUTHOR CONTRIBUTIONS

Xiangyu Zhou conceptualised the study. Xiangyu Zhou performed the data curation, statistical analysis and drafted the manuscript. Jiawen Lu, Zhenqian Wang and Gloria Hoi‐Yee Li were involved in data interpretation and manuscript revision. Kenneth King‐Yip Cheng initiated and supervised the project and revised the manuscript. All authors have reviewed and approved the published version of the manuscript.

## CONFLICT OF INTEREST STATEMENT

No potential conflict of interest relevant to this article was reported.

## Supporting information


**Data S1** Supporting information.

**Data S2** Supplementary tables.

## Data Availability

All publicly available GWAS/GWAS meta‐analysis summary statistics associated with BCAA‐related metabolites (Tables S1, S2) and T2D‐related traits (Table S3) have been listed in detail, and are available for download from the NHGRI‐EBI GWAS catalogue (https://www.ebi.ac.uk/gwas) and the MAGIC consortiums (https://magicinvestigators.org/).

## References

[1] Dludla PV , Mabhida SE , Ziqubu K , et al. Pancreatic β‐cell dysfunction in type 2 diabetes: implications of inflammation and oxidative stress. World J Diabetes. 2023;14(3):130‐146. 37035220 10.4239/wjd.v14.i3.130 PMC10075035

[2] Newgard CB , An J , Bain JR , et al. A branched‐chain amino acid‐related metabolic signature that differentiates obese and lean humans and contributes to insulin resistance. Cell Metab. 2009;9(4):311‐326. 19356713 10.1016/j.cmet.2009.02.002 PMC3640280

[3] Ding C , Egli L , Bosco N , et al. Plasma branched‐chain amino acids are associated with greater fasting and postprandial insulin secretion in non‐diabetic Chinese adults. Front Nutr. 2021;8:664939. 33996878 10.3389/fnut.2021.664939 PMC8113402

[4] Mansoori S , Ho MY , Ng KK , Cheng KK . Branched‐chain amino acid metabolism: pathophysiological mechanism and therapeutic intervention in metabolic diseases. Obes Rev. 2025;26(2):e13856. 39455059 10.1111/obr.13856 PMC11711082

[5] Biswas D , Dao KT , Mercer A , et al. Branched‐chain ketoacid overload inhibits insulin action in the muscle. J Biol Chem. 2020;295(46):15597‐15621. 32878988 10.1074/jbc.RA120.013121 PMC7667962

[6] Bjune MS , Lawrence‐Archer L , Laupsa‐Borge J , et al. Metabolic role of the hepatic valine/3‐hydroxyisobutyrate (3‐HIB) pathway in fatty liver disease. EBioMedicine. 2023;91:104569. 37084480 10.1016/j.ebiom.2023.104569 PMC10148099

[7] Newgard CB . Interplay between lipids and branched‐chain amino acids in development of insulin resistance. Cell Metab. 2012;15(5):606‐614. 22560213 10.1016/j.cmet.2012.01.024 PMC3695706

[8] Yang J , Chi Y , Burkhardt BR , Guan Y , Wolf BA . Leucine metabolism in regulation of insulin secretion from pancreatic beta cells. Nutr Rev. 2010;68(5):270‐279. 20500788 10.1111/j.1753-4887.2010.00282.x PMC2969169

[9] Lotta LA , Scott RA , Sharp SJ , et al. Genetic predisposition to an impaired metabolism of the branched‐chain amino acids and risk of type 2 diabetes: a Mendelian randomisation analysis. PLoS Med. 2016;13(11):e1002179. 27898682 10.1371/journal.pmed.1002179 PMC5127513

[10] Mosley JD , Shi M , Agamasu D , et al. Branched‐chain amino acids and type 2 diabetes: a bidirectional Mendelian randomization analysis. Obesity. 2024;32(2):423‐435. 38269471 10.1002/oby.23951 PMC10827349

[11] Turley P , Walters RK , Maghzian O , et al. Multi‐trait analysis of genome‐wide association summary statistics using MTAG. Nat Genet. 2018;50(2):229‐237. 29292387 10.1038/s41588-017-0009-4 PMC5805593

[12] Karjalainen MK , Karthikeyan S , Oliver‐Williams C , et al. Genome‐wide characterization of circulating metabolic biomarkers. Nature. 2024;628(8006):130‐138. 38448586 10.1038/s41586-024-07148-y PMC10990933

[13] Richardson TG , Leyden GM , Wang Q , et al. Characterising metabolomic signatures of lipid‐modifying therapies through drug target mendelian randomisation. PLoS Biol. 2022;20(2):e3001547. 35213538 10.1371/journal.pbio.3001547 PMC8906647

[14] Chen Y , Lu T , Pettersson‐Kymmer U , et al. Genomic atlas of the plasma metabolome prioritizes metabolites implicated in human diseases. Nat Genet. 2023;55(1):44‐53. 36635386 10.1038/s41588-022-01270-1 PMC7614162

[15] Shin S‐Y , Fauman EB , Petersen A‐K , et al. An atlas of genetic influences on human blood metabolites. Nat Genet. 2014;46(6):543‐550. 24816252 10.1038/ng.2982 PMC4064254

[16] Willer CJ , Li Y , Abecasis GR . METAL: fast and efficient meta‐analysis of genomewide association scans. Bioinformatics. 2010;26(17):2190‐2191. 20616382 10.1093/bioinformatics/btq340 PMC2922887

[17] Xiao L , Liu S , Wu Y , et al. The interactions between host genome and gut microbiome increase the risk of psychiatric disorders: Mendelian randomization and biological annotation. Brain Behav Immun. 2023;113:389‐400. 37557965 10.1016/j.bbi.2023.08.003 PMC11258998

[18] Bowden J , Holmes MV . Meta‐analysis and Mendelian randomization: a review. Res Synth Methods. 2019;10(4):486‐496. 30861319 10.1002/jrsm.1346 PMC6973275

[19] Sanna S , van Zuydam NR , Mahajan A , et al. Causal relationships among the gut microbiome, short‐chain fatty acids and metabolic diseases. Nat Genet. 2019;51(4):600‐605. 30778224 10.1038/s41588-019-0350-x PMC6441384

[20] Burgess S . Sample size and power calculations in Mendelian randomization with a single instrumental variable and a binary outcome. Int J Epidemiol. 2014;43(3):922‐929. 24608958 10.1093/ije/dyu005 PMC4052137

[21] Burgess S , Butterworth A , Thompson SG . Mendelian randomization analysis with multiple genetic variants using summarized data. Genet Epidemiol. 2013;37(7):658‐665. 24114802 10.1002/gepi.21758 PMC4377079

[22] Bowden J , Davey Smith G , Haycock PC , Burgess S . Consistent estimation in Mendelian randomization with some invalid instruments using a weighted median estimator. Genet Epidemiol. 2016;40(4):304‐314. 27061298 10.1002/gepi.21965 PMC4849733

[23] Burgess S , Foley CN , Allara E , Staley JR , Howson JM . A robust and efficient method for Mendelian randomization with hundreds of genetic variants. Nat Commun. 2020;11(1):376. 31953392 10.1038/s41467-019-14156-4 PMC6969055

[24] Bowden J , Davey Smith G , Burgess S . Mendelian randomization with invalid instruments: effect estimation and bias detection through Egger regression. Int J Epidemiol. 2015;44(2):512‐525. 26050253 10.1093/ije/dyv080 PMC4469799

[25] Verbanck M , Chen CY , Neale B , Do R . Detection of widespread horizontal pleiotropy in causal relationships inferred from Mendelian randomization between complex traits and diseases. Nat Genet. 2018;50(5):693‐698. 29686387 10.1038/s41588-018-0099-7 PMC6083837

[26] Skrivankova VW , Richmond RC , Woolf BA , et al. Strengthening the reporting of observational studies in epidemiology using Mendelian randomization: the STROBE‐MR statement. JAMA. 2021;326(16):1614‐1621. 34698778 10.1001/jama.2021.18236

[27] Yang C , Fagan AM , Perrin RJ , Rhinn H , Harari O , Cruchaga C . Mendelian randomization and genetic colocalization infer the effects of the multi‐tissue proteome on 211 complex disease‐related phenotypes. Genome Med. 2022;14(1):140. 36510323 10.1186/s13073-022-01140-9 PMC9746220

[28] Zuber V , Grinberg NF , Gill D , et al. Combining evidence from Mendelian randomization and colocalization: review and comparison of approaches. Genome Med. 2022;14(1):140. 35452592 10.1016/j.ajhg.2022.04.001 PMC7612737

[29] Dong Y , Hu A‐q , Han B‐x , et al. Mendelian randomization analysis reveals causal effects of blood lipidome on gestational diabetes mellitus. Cardiovasc Diabetol. 2024;23(1):335. 39261922 10.1186/s12933-024-02429-2 PMC11391602

[30] Fan Q , Wen S , Zhang Y , et al. Assessment of circulating proteins in thyroid cancer: proteome‐wide Mendelian randomization and colocalization analysis. iScience. 2024;27(6):109961. 38947504 10.1016/j.isci.2024.109961 PMC11214373

[31] Davyson E , Shen X , Gadd DA , et al. Metabolomic investigation of major depressive disorder identifies a potentially causal association with polyunsaturated fatty acids. Biol Psychiatry. 2023;94(8):630‐639. 36764567 10.1016/j.biopsych.2023.01.027 PMC10804990

[32] Lotta LA , Pietzner M , Stewart ID , et al. A cross‐platform approach identifies genetic regulators of human metabolism and health. Nat Genet. 2021;53(1):54‐64. 33414548 10.1038/s41588-020-00751-5 PMC7612925

[33] Karusheva Y , Koessler T , Strassburger K , et al. Short‐term dietary reduction of branched‐chain amino acids reduces meal‐induced insulin secretion and modifies microbiome composition in type 2 diabetes: a randomized controlled crossover trial. Am J Clin Nutr. 2019;110(5):1098‐1107. 31667519 10.1093/ajcn/nqz191 PMC6821637

[34] Wilson JM , Lowery RP , Joy JM , et al. The effects of 12 weeks of beta‐hydroxy‐beta‐methylbutyrate free acid supplementation on muscle mass, strength, and power in resistance‐trained individuals: a randomized, double‐blind, placebo‐controlled study. Eur J Appl Physiol. 2014;114(6):1217‐1227. 24599749 10.1007/s00421-014-2854-5 PMC4019830

[35] Manzano M , Girón MD , Salto R , et al. Arginine and lysine supplementation potentiates the beneficial β‐hydroxy ß‐methyl butyrate (HMB) effects on skeletal muscle in a rat model of diabetes. Nutrients. 2023;15(22):4706. 38004100 10.3390/nu15224706 PMC10674618

[36] Schadock I , Freitas BG , Moreira IL , et al. Supplementation with beta‐hydroxy‐beta‐methylbutyrate impacts glucose homeostasis and increases liver size in trained mice. Int J Vitam Nutr Res. 2020;90(1‐2):113‐123. 30545278 10.1024/0300-9831/a000445

[37] Sharawy MH , El‐Awady MS , Megahed N , Gameil NM . The ergogenic supplement β‐hydroxy‐β‐methylbutyrate (HMB) attenuates insulin resistance through suppressing GLUT‐2 in rat liver. Can J Physiol Pharmacol. 2016;94(5):488‐497. 26871756 10.1139/cjpp-2015-0385

[38] Cheng Q , Beltran VD , Chan SM , Brown JR , Bevington A , Herbert TP . System‐L amino acid transporters play a key role in pancreatic β‐cell signalling and function. J Mol Endocrinol. 2016;56(3):175‐187. 26647387 10.1530/JME-15-0212

[39] McCormack SE , Shaham O , McCarthy MA , et al. Circulating branched‐chain amino acid concentrations are associated with obesity and future insulin resistance in children and adolescents. Pediatr Obes. 2013;8(1):52‐61. 22961720 10.1111/j.2047-6310.2012.00087.x PMC3519972

[40] Wang TJ , Larson MG , Vasan RS , et al. Metabolite profiles and the risk of developing diabetes. Nat Med. 2011;17(4):448‐453. 21423183 10.1038/nm.2307 PMC3126616

[41] Roberts LD , Boström P , O'Sullivan JF , et al. Β‐aminoisobutyric acid induces browning of white fat and hepatic β‐oxidation and is inversely correlated with cardiometabolic risk factors. Cell Metab. 2014;19(1):96‐108. 24411942 10.1016/j.cmet.2013.12.003 PMC4017355

[42] Rietman A , Stanley TL , Clish C , et al. Associations between plasma branched‐chain amino acids, β‐aminoisobutyric acid and body composition. J Nutr Sci. 2016;5:e6. 27313851 10.1017/jns.2015.37 PMC4791517

[43] Faiz H , Heiston EM , Malin SK . Β‐aminoisobutyric acid relates to favorable glucose metabolism through adiponectin in adults with obesity independent of prediabetes. J Diabetes Res. 2023;2023(1):4618215. 37780967 10.1155/2023/4618215 PMC10539091

[44] Zhou M , Shao J , Wu C‐Y , et al. Targeting BCAA catabolism to treat obesity‐associated insulin resistance. Diabetes. 2019;68(9):1730‐1746. 31167878 10.2337/db18-0927 PMC6702639

[45] Shin AC , Fasshauer M , Filatova N , et al. Brain insulin lowers circulating BCAA levels by inducing hepatic BCAA catabolism. Cell Metab. 2014;20(5):898‐909. 25307860 10.1016/j.cmet.2014.09.003 PMC4254305

[46] Wang Q , Holmes MV , Davey Smith G , Ala‐Korpela M . Genetic support for a causal role of insulin resistance on circulating branched‐chain amino acids and inflammation. Diabetes Care. 2017;40(12):1779‐1786. 29046328 10.2337/dc17-1642 PMC5701741

[47] Mahendran Y , Jonsson A , Have CT , et al. Genetic evidence of a causal effect of insulin resistance on branched‐chain amino acid levels. Diabetologia. 2017;60(5):873‐878. 28184960 10.1007/s00125-017-4222-6

[48] Lian K , Du C , Liu Y , et al. Impaired adiponectin signaling contributes to disturbed catabolism of branched‐chain amino acids in diabetic mice. Diabetes. 2015;64(1):49‐59. 25071024 10.2337/db14-0312

[49] Andrade ML , Gilio GR , Perandini LA , et al. PPARγ‐induced upregulation of subcutaneous fat adiponectin secretion, glyceroneogenesis and BCAA oxidation requires mTORC1 activity. Biochim Biophys Acta Mol Cell Biol Lipids. 2021;1866(8):158967. 34004356 10.1016/j.bbalip.2021.158967 PMC9391032

[50] Mootha VK , Lindgren CM , Eriksson K‐F , et al. PGC‐1α‐responsive genes involved in oxidative phosphorylation are coordinately downregulated in human diabetes. Nat Genet. 2003;34(3):267‐273. 12808457 10.1038/ng1180

[51] Benton C , Holloway G , Han X‐X , et al. Increased levels of peroxisome proliferator‐activated receptor gamma, coactivator 1 alpha (PGC‐1α) improve lipid utilisation, insulin signalling and glucose transport in skeletal muscle of lean and insulin‐resistant obese Zucker rats. Diabetologia. 2010;53(9):2008‐2019. 20490453 10.1007/s00125-010-1773-1

[52] Sjögren RJ , Rizo‐Roca D , Chibalin AV , et al. Branched‐chain amino acid metabolism is regulated by ERRα in primary human myotubes and is further impaired by glucose loading in type 2 diabetes. Diabetologia. 2021;64(9):2077‐2091. 34131782 10.1007/s00125-021-05481-9 PMC8382616

[53] Agus A , Clément K , Sokol H . Gut microbiota‐derived metabolites as central regulators in metabolic disorders. Gut. 2021;70(6):1174‐1182. 33272977 10.1136/gutjnl-2020-323071 PMC8108286

[54] Vanweert F , Neinast M , Tapia EE , et al. A randomized placebo‐controlled clinical trial for pharmacological activation of BCAA catabolism in patients with type 2 diabetes. Nat Commun. 2022;13(1):3508. 35717342 10.1038/s41467-022-31249-9 PMC9206682

[55] Roth Flach RJ , Bollinger E , Reyes AR , et al. Small molecule branched‐chain ketoacid dehydrogenase kinase (BDK) inhibitors with opposing effects on BDK protein levels. Nat Commun. 2023;14(1):4812. 37558654 10.1038/s41467-023-40536-y PMC10412597

[56] Hiraiwa H , Okumura T , Kondo T , et al. Usefulness of the plasma branched‐chain amino acid/aromatic amino acid ratio for predicting future cardiac events in patients with heart failure. J Cardiol. 2020;75(6):689‐696. 32001073 10.1016/j.jjcc.2019.12.016

[57] Hiraiwa H , Okumura T , Kondo T , et al. Prognostic value of leucine/phenylalanine ratio as an amino acid profile of heart failure. Heart Vessel. 2021;36(7):965‐977. 10.1007/s00380-020-01765-z 33481086

[58] Hiraiwa H , Okumura T , Murohara T . Amino acid profiling to predict prognosis in patients with heart failure: an expert review. ESC Heart Fail. 2023;10(1):32‐43. 36300549 10.1002/ehf2.14222 PMC9871678

[59] Kimura Y , Okumura T , Kazama S , et al. Usefulness of plasma branched‐chain amino acid analysis in predicting outcomes of patients with nonischemic dilated cardiomyopathy. Int Heart J. 2020;61(4):739‐747. 32684600 10.1536/ihj.20-010

