# Supplementary material for: Causal role of genetically predicted impairment of branched‐chain amino acid catabolism on insulin secretion and insulin resistance in type 2 diabetes
Source: Diabetes Obes Metab. 2026 Feb 18;28(5):3849–60. doi: 10.1111/dom.70565 (PMC13071227; doi:10.1111/dom.70565)
Supplement: Supplementary file 1 — Data S1 Supporting information. [file DOM-28-3849-s001.docx]

**Supplementary Information**

**Supplementary methods**

1. GWAS meta-analysis
2. Multi-trait analysis of GWAS (MTAG)
3. Functional Mapping and Annotation (FUMA)
4. Mendelian randomization (MR) analysis
   1. Selection of instrumental variables (IVs)
   2. MR analysis
5. Statistical analysis

**Supplementary Figures**

**Figure S1.** The BCAA catabolism pathways.

**Figure S2.** Manhattan plots of GWAS meta-analysis (left) and MTAG results (right) of each BCAA.

**Figure S3.** Quantile-Quantile (QQ) plots for GWAS meta-analysis (left) and MTAG results (right) of leucine, valine and isoleucine.

**Figure S4.** Inadequately powered Mendelian randomization analysis of BCAAs and T2D-related traits.

**Figure S5.** Inadequately powered Mendelian randomization analysis of BCAA catabolites and T2D-related traits.

**Figure S6.** Boxplots of leave-one-out analysis for statistically or suggestively significant associations.

**Supplementary methods**

1. **GWAS meta-analysis**

METAL^1^ was used to conduct all meta-analyses of genome-wide association studies (GWAS), with effect size weights estimated based on standard error. SNPs with significant heterogeneity (*p*<0.001) and a minor allele frequency (MAF)<0.01 were excluded from meta-analysis. The genomic inflation factor (λ_GC_) and linkage disequilibrium score regression (LDSC) intercepts were used to assess the inflation of genetic signals based on the European dataset from Phase 3 of the 1000 Genomes Project^2^, which were compared to determine whether the observed inflation was caused by polygenicity or population stratification (confounding)^3^.

1. **Multi-trait analysis of GWAS (MTAG)**

MTAG jointly analyses genetically correlated traits using GWAS summary statistics to improve power for trait-specific effect estimation^4^. The mean chi-square (χ²) is the genome-wide average of single-SNP association χ² statistics; under standard polygenic assumptions, it was expected to increase with sample size and SNP-heritability. A mean χ²<1.02 suggests an underpowered input GWAS^5^. As MTAG’s trait-specific false discovery can become substantial when it is applied to a large number of low-powered GWAS or GWAS with large difference in statistical power, we restricted MTAG to traits with mean χ²≥1.02 to help mitigate potential inflation of trait-specific false positives^5^.

1. **Functional Mapping and Annotation (FUMA)**

FUMA defined independent variants as those reaching genome-wide significance (GWAS *p*<5×10⁻⁸, MAF≥0.01) yet not in high linkage disequilibrium (LD) with each other (*r^2^*<0.6, European reference panel of the 1000 Genomes [1000G] Project Phase 3). Lead variants were selected with *p*<5×10^-8^ and *r^2^*<0.1. Genomic risk loci were defined by merging LD blocks around independent significant variants or lead variants if the blocks were within 250 kb of each other.

1. **Mendelian randomization (MR) analysis**

**4.1 Selection of instrumental variables (IVs)**

Independently significant variants were initially selected as IVs for the exposure after LD clumping based on the European reference panel of the 1000G Project. If a particular IV was not present in the outcome GWAS, proxies were used by LD tagging (*r^2^*≥0.8). Palindromic and ambiguous SNPs were excluded from the analysis if nonpalindromic proxies in high LD (*r²*≥0.8) could not be identified. The strength of selected IVs was measured by F-statistics, which evaluate the proportion of variance explained by the IVs relative to the sample size and number of variables^6^. IVs with F<10 were removed to minimize potential bias from weak IVs^6^. If an IV had a genome-wide significant association with body mass index (BMI) and waist-hip ratio (WHR), this IV was excluded due to the possible violation of the independence and exclusion assumption.

**4.2 MR analysis**

All the IVs were oriented such that the effect alleles were positively associated with the exposure. Effect alleles were harmonized across the summary data of the exposure and outcome dataset to ensure consistency. The causal direction of each IV was tested using Steiger filtering^7^. The IVs were excluded if the variance explained in the outcome was more than the exposure.

We calculated the ratio of the size of each variant’s effect on outcome divided by the size of its effect on the exposure, known as Wald ratios^8^. The Inverse-Variance Weighted (IVW) method combines the Wald ratios of each variant into a weighted average of the individual ratios, which assigns greater weight to values with higher precision or lower variance (i.e., estimates are weighed by the inverse of their variance)^9^. The IVW method is the main MR method that assumes that all instrumental variables (IVs) are valid. However, the method is very sensitive to violations of these assumptions, e.g. even with an invalid IV^9^. Furthermore, heterogeneity among the estimates needs to be assessed before weighted averaging the Wald ratios. Moderate heterogeneity may arise from sampling error, but excessive heterogeneity may suggest that the IVs are invalid, leading to causal estimation bias^8^. Heterogeneity can result from both biological causes (e.g., different genetic variants affect BMI differently) and non-biological or non-causal factors (e.g., confounding due to horizontal pleiotropy). Commonly used statistical methods include the Q statistic, which tests whether heterogeneity exceeds chance error, and the *I²* statistic, which quantifies the proportion of heterogeneity in the total variation^10^. The *P_heterogeneity_* >0.05 indicates no heterogeneity^10^.

To prove the robustness of our findings, several sensitivity analyses were conducted. The weighted median method estimates causal effects as the weighted median of individual SNP ratio estimates, which gives consistent results even with up to 50% of invalid instruments^11^. While this method is more robust than IVW to invalid IVs, it is less efficient when all IVs are valid and may be biased if more than 50% of the instruments violate the assumptions^11^. The contamination mixture (ConMix) method models the Wald ratio estimates of all instruments as a normal mixture distribution and automatically identifies the largest cluster to represent the true causal effect^12^. ConMix demonstrates strong robustness when most IVs are effective, effectively reducing the impact of partially ineffective instruments. ConMix method had the lowest mean square error in detecting causal effects despite invalid IVs^12^. However, when the ratio estimates of IVs show highly overlapping distributions or most IVs are ineffective, ConMix cannot accurately distinguish between effective and ineffective instruments, leading to reduced precision in main effect estimates or even bias^12^. MR-Egger regression is used to address horizontal pleiotropy by fitting a weighted linear regression of SNP-outcome associations and SNP-exposure associations, allowing the intercept term to be non-zero^13^. The slope provides the causal estimate, while the intercept tests for and quantifies average directional pleiotropy across all SNPs^13^. This method has advantages in detecting and adjusting pleiotropy but has lower statistical power to detect causal effects, is sensitive to measurement error, and requires meeting the InSIDE (Instrument Strength Independent of Direct Effect, i.e., the pleiotropy effect is independent of the influence of SNP on exposure) assumption. The presence of horizontal pleiotropy was indicated by *p*<0.05 in MR-Egger intercept test^13^. Finally, MR-PRESSO (Pleiotropy RESidual Sum and Outlier) can detect and correct horizontal pleiotropy by identifying outlier SNPs that deviate from the expected MR relationship^14^. MR-PRESSO worked best if horizontal pleiotropy was present in less than half of the IVs^14^. The MR-PRESSO global test was used to assess the total horizontal pleiotropy of all the IVs, while the MR-PRESSO outlier test removed pleiotropic outliers and corrected the causal estimates for horizontal pleiotropy^14^. MR-PRESSO enhances robustness against pleiotropic bias but may fail to detect subtle forms of pleiotropy and relies on accurate detection of outliers^14^.

Therefore, we supplemented the causal estimates of IVW with methods such as weighted median, MR-Egger, ConMix, and MR-PRESSO, which effectively address ineffective or pleiotropic IVs and improve the reliability and robustness of the results.

1. **Statistical analysis**

The IVW, Cochran’s Q, weighted median, and MR-Egger analyses were conducted using the “TwoSampleMR” package (v0.5.6)^15^. ConMix and MR-PRESSO analyses were performed using the “MendelianRandomization” (v0.10.0)^16^ and “MRPRESSO” (v.1.0)^14^ packages, respectively. The online tool (<https://sb452.shinyapps.io/power/>)^17^ was used to calculate the statistical power for each MR analysis. Power was calculated assuming a two-sided α=0.05 and a minimum detectable effect of β = 0.10 (per 1-SD increase in the exposure).Colocalization analyses were performed using coloc R package (v.5.2.3)^18^.

**Supplementary Figures**

**
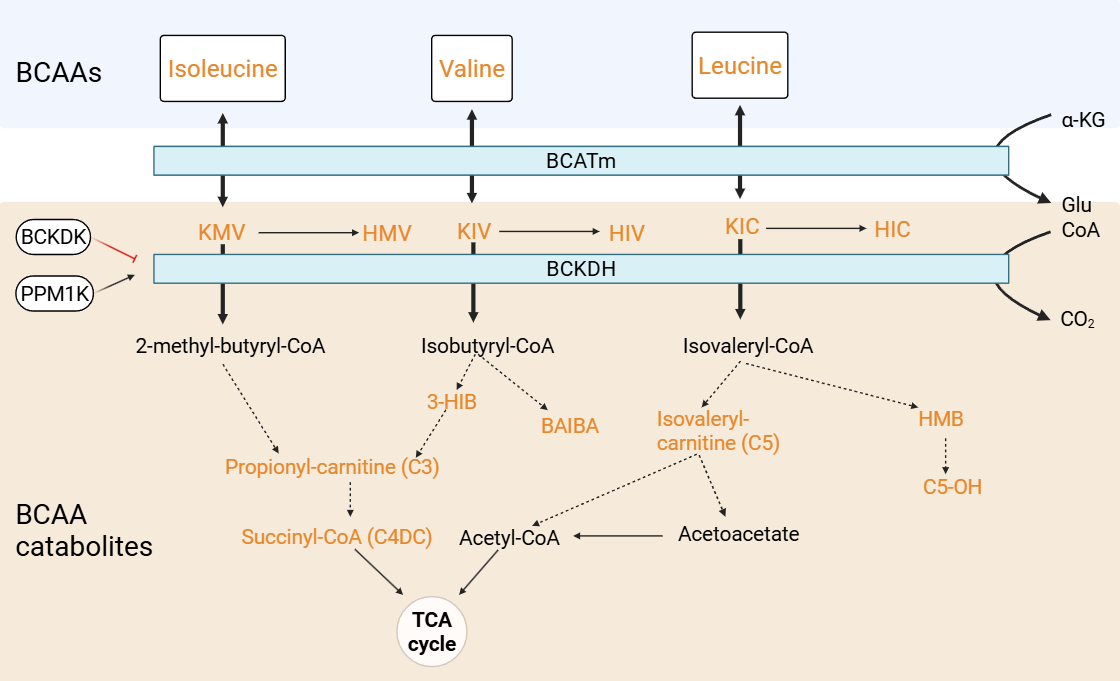
**

**Figure S1. The BCAA catabolism pathways.** The BCAA metabolites (including BCAAs and BCAA catabolites) analyzed in this study are highlighted in orange text. C3 and C4DC are common downstream metabolites shared by the catabolic pathways of isoleucine and valine. BCAAs undergo reversible transamination catalyzed by mitochondrial or cell membrane branched-chain aminotransferase (BCATm) isoenzymes to produce the branched-chain keto acids (BCKAs), including 4-methyl-2-oxopentanoic acid (KIC), 3-methyl-2-oxobutanoic acid (KIV), and 3-methyl-2-oxopentanoatic acid (KMV). KIC, KIV and KMV could be reduced and produce the hydroxyl derivatives α-hydroxyisocaproate (HIC) ^19,20^, α-hydroxyisovalerate (HIV) ^19-21^, 2-hydroxy-3-methylvalerate (HMV) ^20,22^, respectively. Furthermore, these BCKAs also undergo irreversible decarboxylation catalyzed by the branched-chain α-ketoacid dehydrogenase complex (BCKDH) to produce the corresponding Acyl-CoA derivates, which enter their respective metabolic pathways. 3-HIB: 3-hydroxyisobutyrate; BAIBA: β-aminoisobutyric acid; HMB: β-hydroxyisovalerate; C5-OH: β-hydroxyisovaleroylcarnitine; α-KG: α-ketoglutarate; Glu: Glutamate; BCKDK: Branched chain ketoacid dehydrogenase kinase; Protein Phosphatase, Mg^2+^/Mn^2+^ Dependent 1K: PPM1K; TCA: Tricarboxylic Acid. Created in BioRender.com


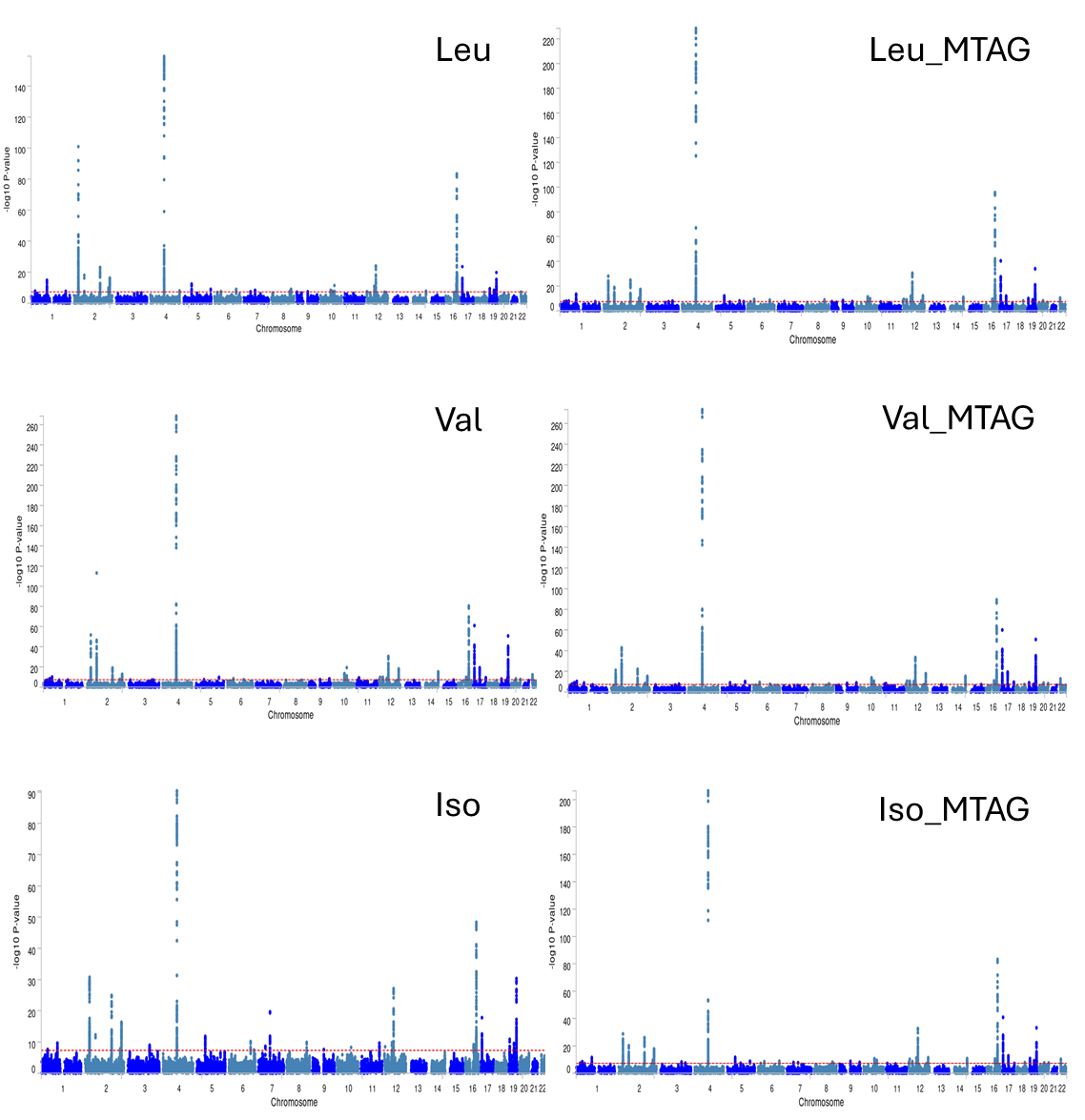


**Figure S2. Manhattan plots of GWAS meta-analysis (left) and MTAG results (right) of each BCAA.** The X-axis represents the chromosomal position, while the Y-axis shows the negative log 10 transformed P-values (−log10(P)) for each SNP. The red dashed line indicates genome-wide significance (*P* = 5 × 10^-8^).


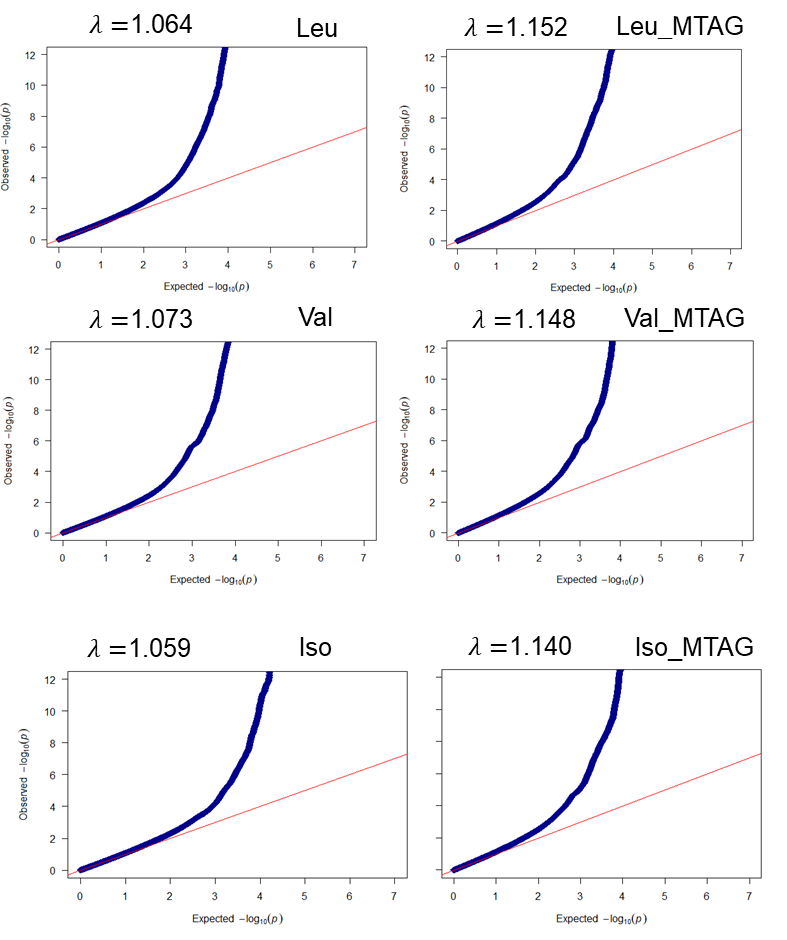


**Figure S3.** Quantile-Quantile (QQ) plots for GWAS meta-analysis (left) and MTAG results (right) of leucine, valine and isoleucine. The genomic inflation factor (lambda: $\lambda$) was shown in the plot. The X-axis and Y-axis show -log10 transformed expected *p-values* and observed *p-values*, respectively. Red line represents the expected distribution of *p-values* under the null hypothesis of no association.


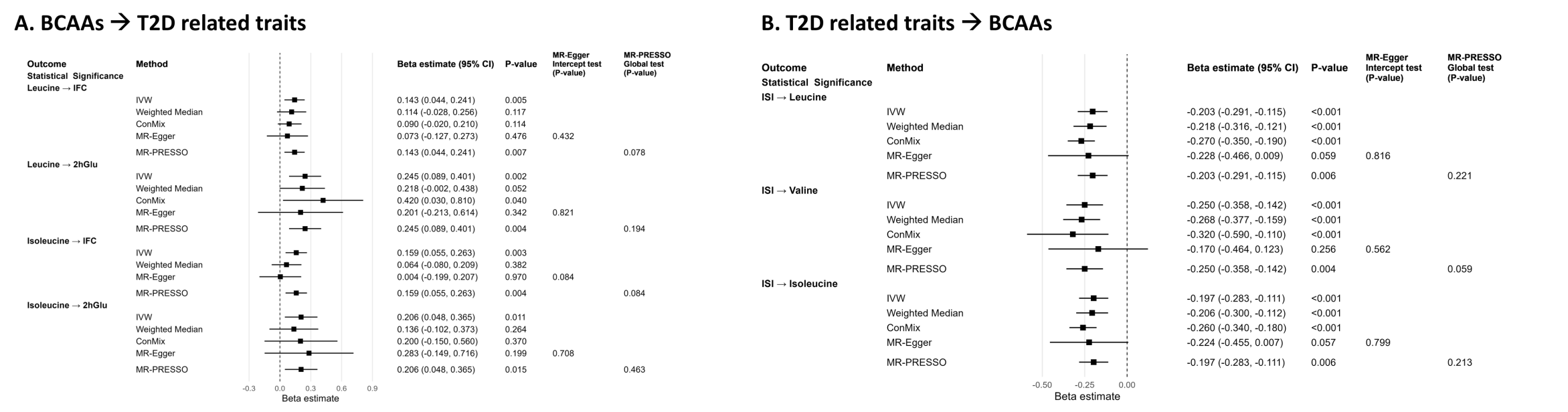


**Figure S4. Inadequately powered Mendelian randomization analysis of BCAAs and T2D-related traits.** MR analyses of power <80% that met statistical or suggestive significance thresholds in (A) forward and (B) reverse directions were presented. For each exposure–outcome pair, causal effect estimates (β) and 95% confidence intervals (CIs) are shown for the primary inverse variance weighted (IVW) method and sensitivity analyses, including weighted median, contamination mixture (ConMix), MR-Egger, and MR Pleiotropy RESidual Sum and Outlier (MR-PRESSO). Horizontal pleiotropy was assessed using the MR-Egger intercept test and the MR-PRESSO global test.

The ConMix result for the association between isoleucine and IFC is not shown in the forest plot because the method does not provide standard p-values and may yield both primary and alternative estimates. Full ConMix results are provided in Table S8b.

IFC: Insulin Fold Change; 2hGlu: 2h Glucose level after an Oral Glucose Challenge Test, Glucose Tolerance; ISI: Modified Stumvoll Insulin Sensitivity Index.


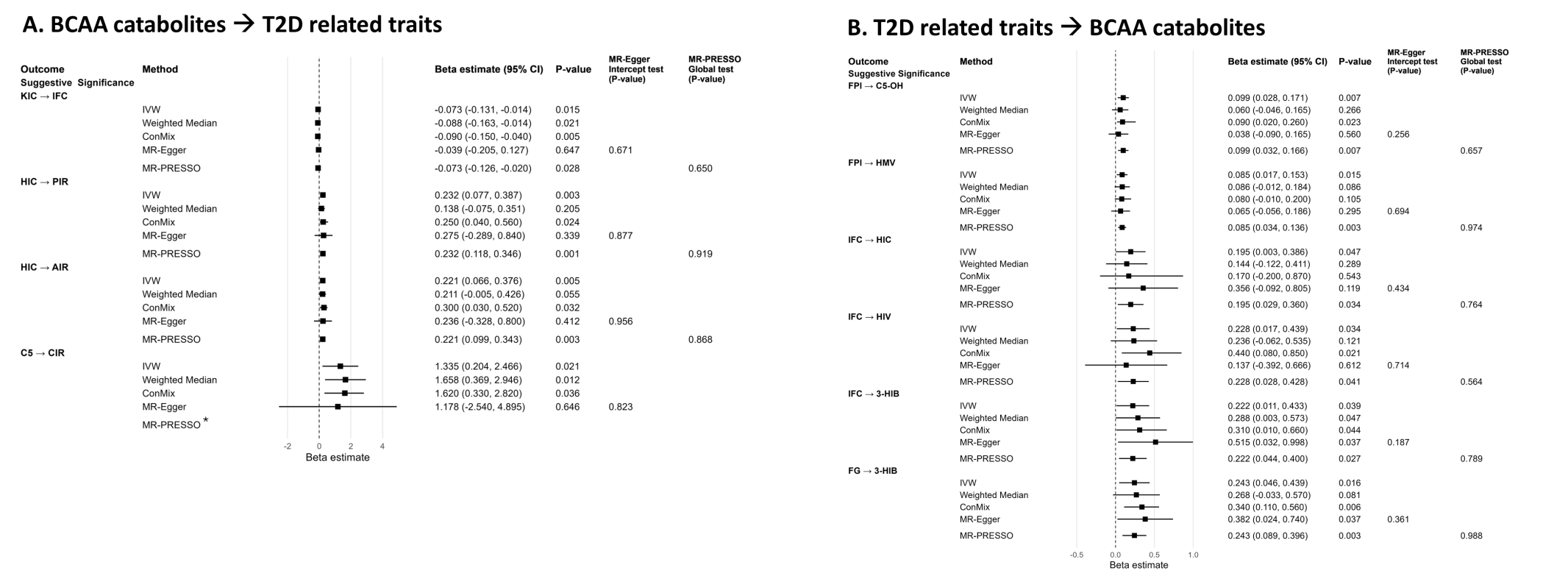


**Figure S5. Bidirectional Mendelian randomization between BCAA catabolites and T2D-related traits.** The forest plots summarize inadequately powered MR results (power <80%) assessing causal relationships between genetically predicted BCAA catabolites and T2D-related traits in (A) forward and (B) reverse directions. Effect sizes (β) and 95% CIs are shown for inverse variance weighted (IVW) as the primary method, alongside sensitivity analyses using weighted median, ConMix, MR-Egger, and MR-PRESSO. Evidence of horizontal pleiotropy was examined via the MR-Egger intercept test and the MR-PRESSO global test.

*For C5–>CIR, MR-PRESSO could not be conducted (due to 3IVs only).

IFC: Insulin Fold Change; 2hGlu: 2h Glucose level after an Oral Glucose Challenge Test, Glucose Tolerance; ISI: Modified Stumvoll Insulin Sensitivity Index; PIR: Peak Insulin Response; AIR: Acute Insulin Response; CIR: Corrected Insulin Response; FPI: Fasting Proinsulin; FG: Fasting Glucose; KIC: 4-methyl-2-oxopentanoate; C5: isovaleryl-carnitine; C5-OH: β-hydroxyisovaleroylcarnitine; HMV: 2-hydroxy-3-methylvalerate; HIC: α-hydroxyisocaproic acid; HIV: α-hydroxyisovalerate; 3-HIB: 3-hydroxyisobutyrate.


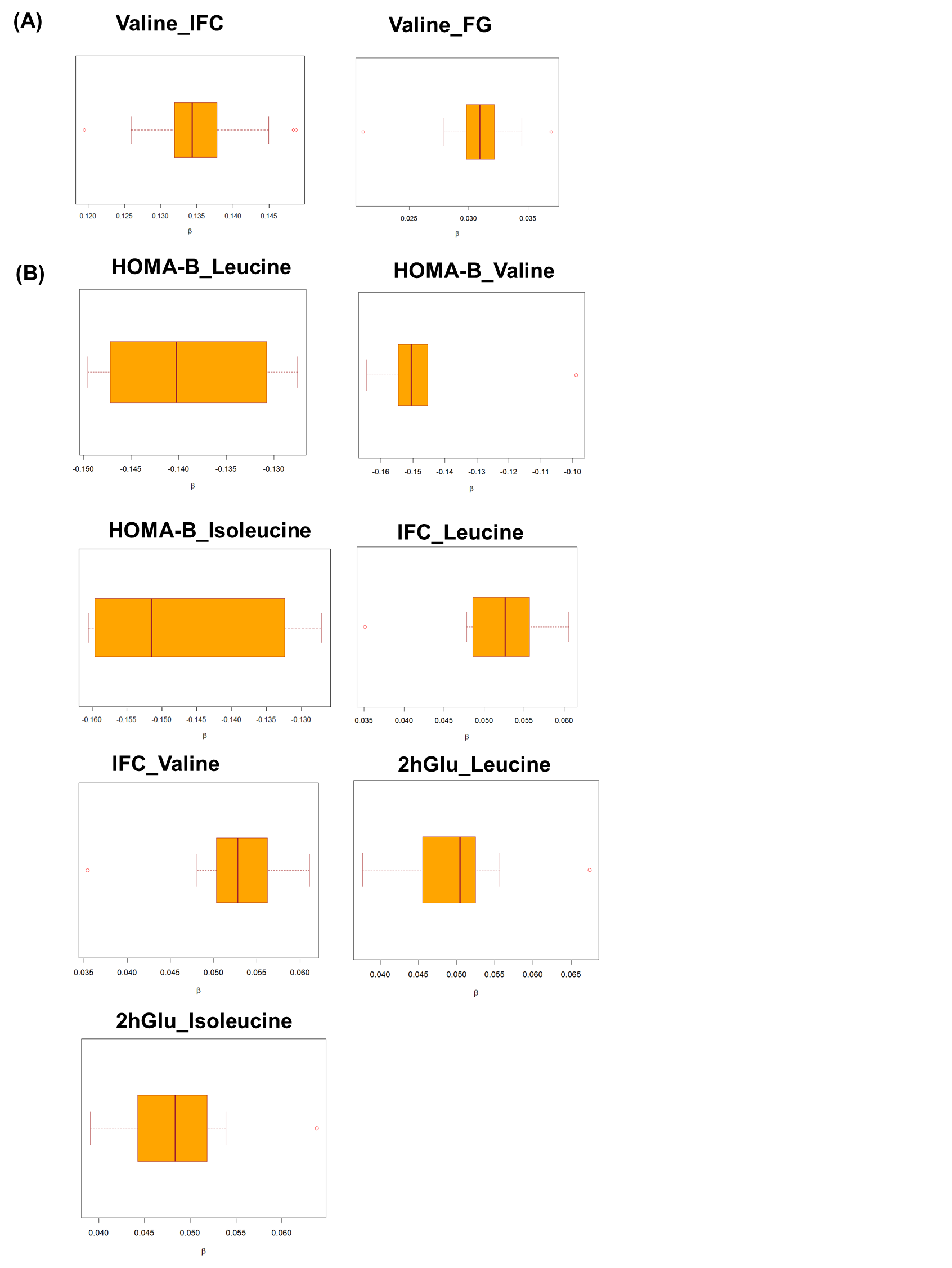


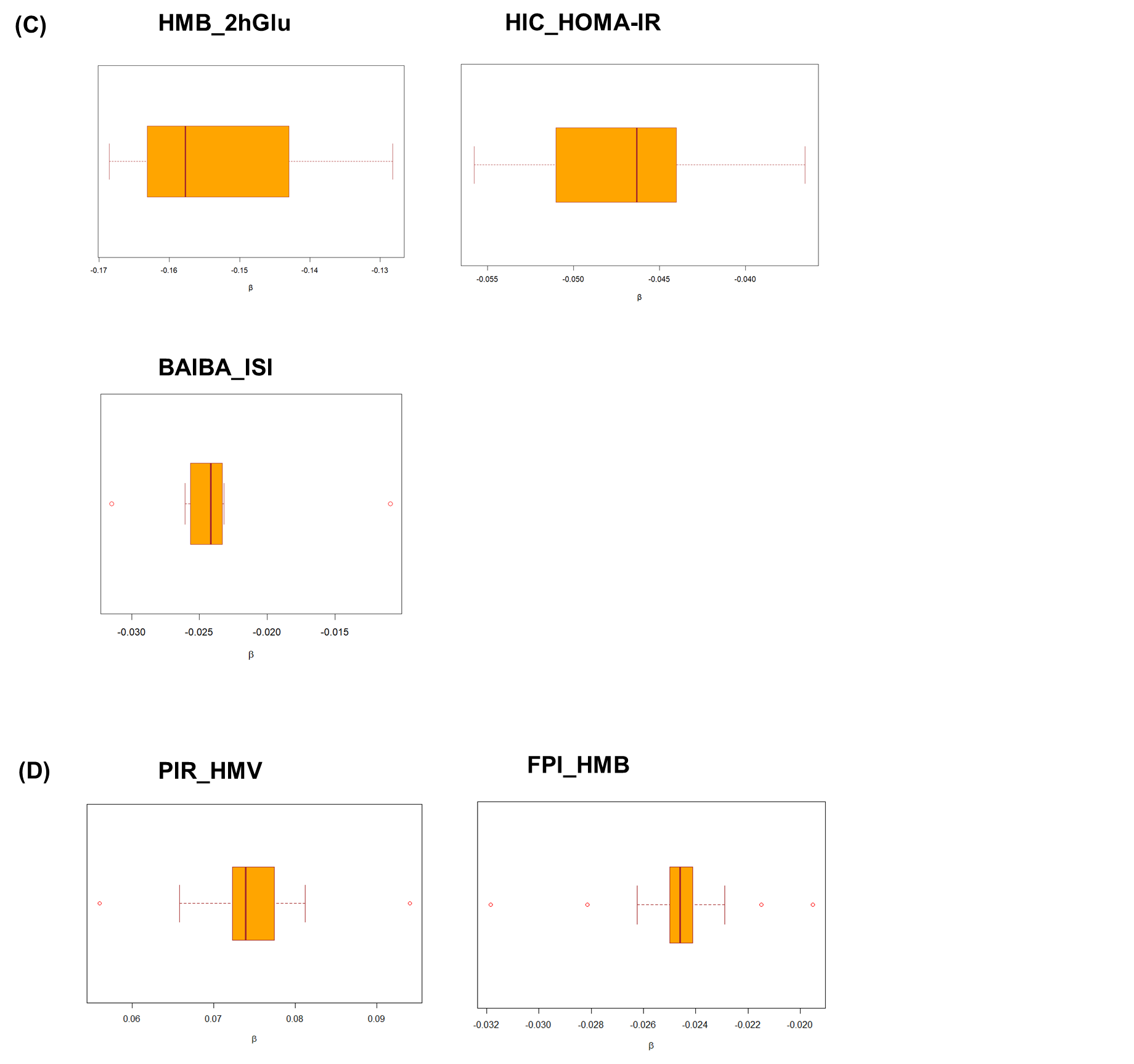


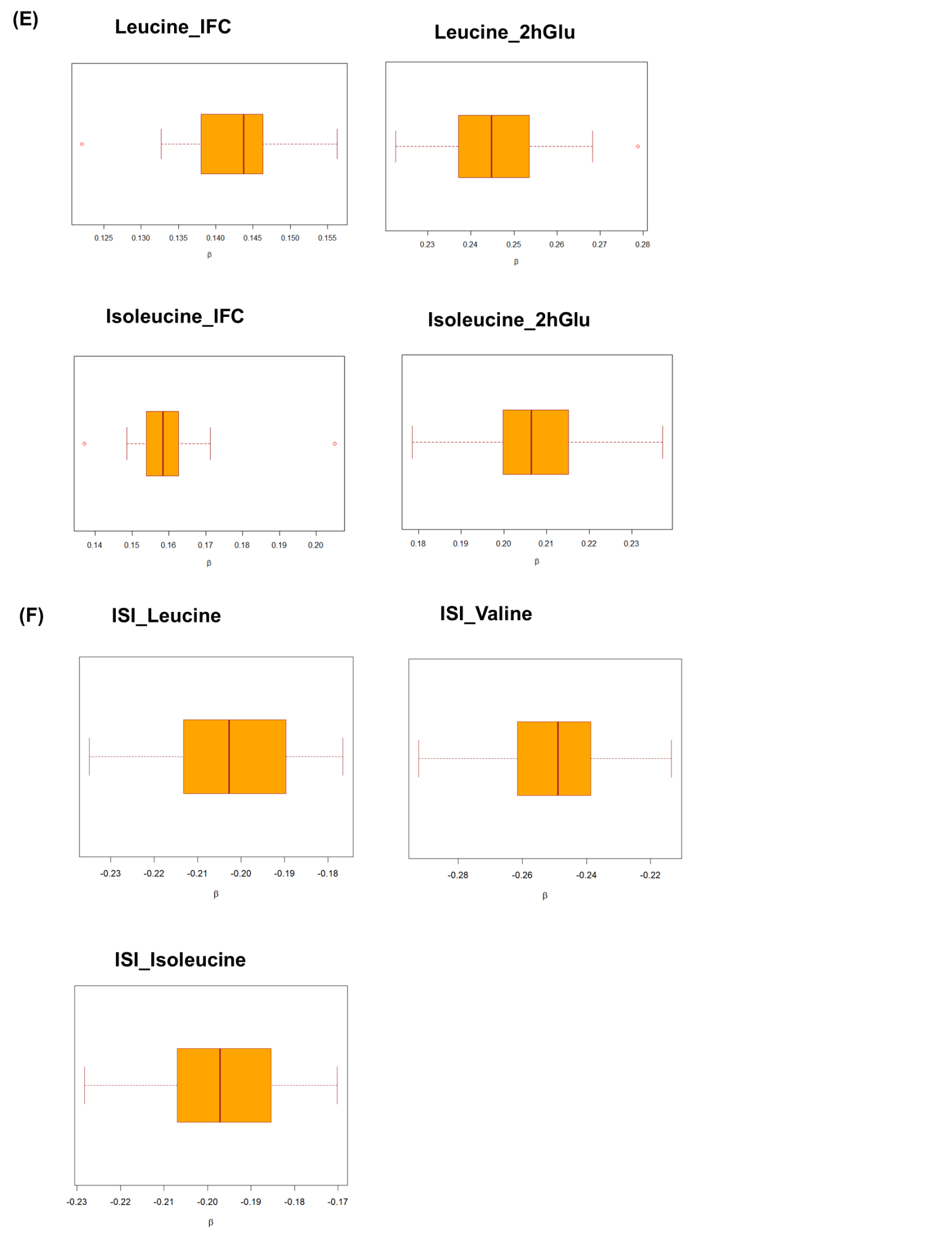


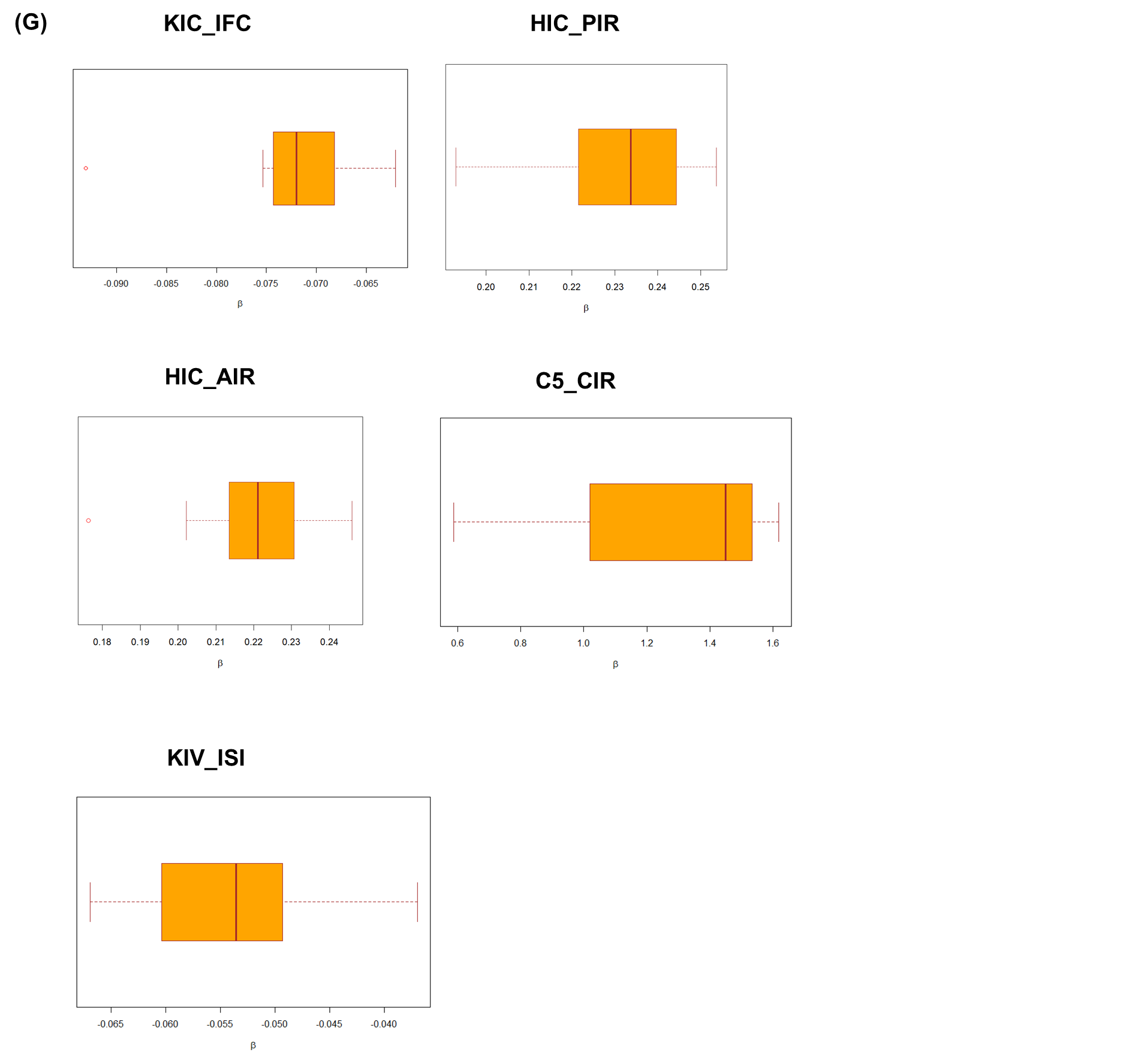


**
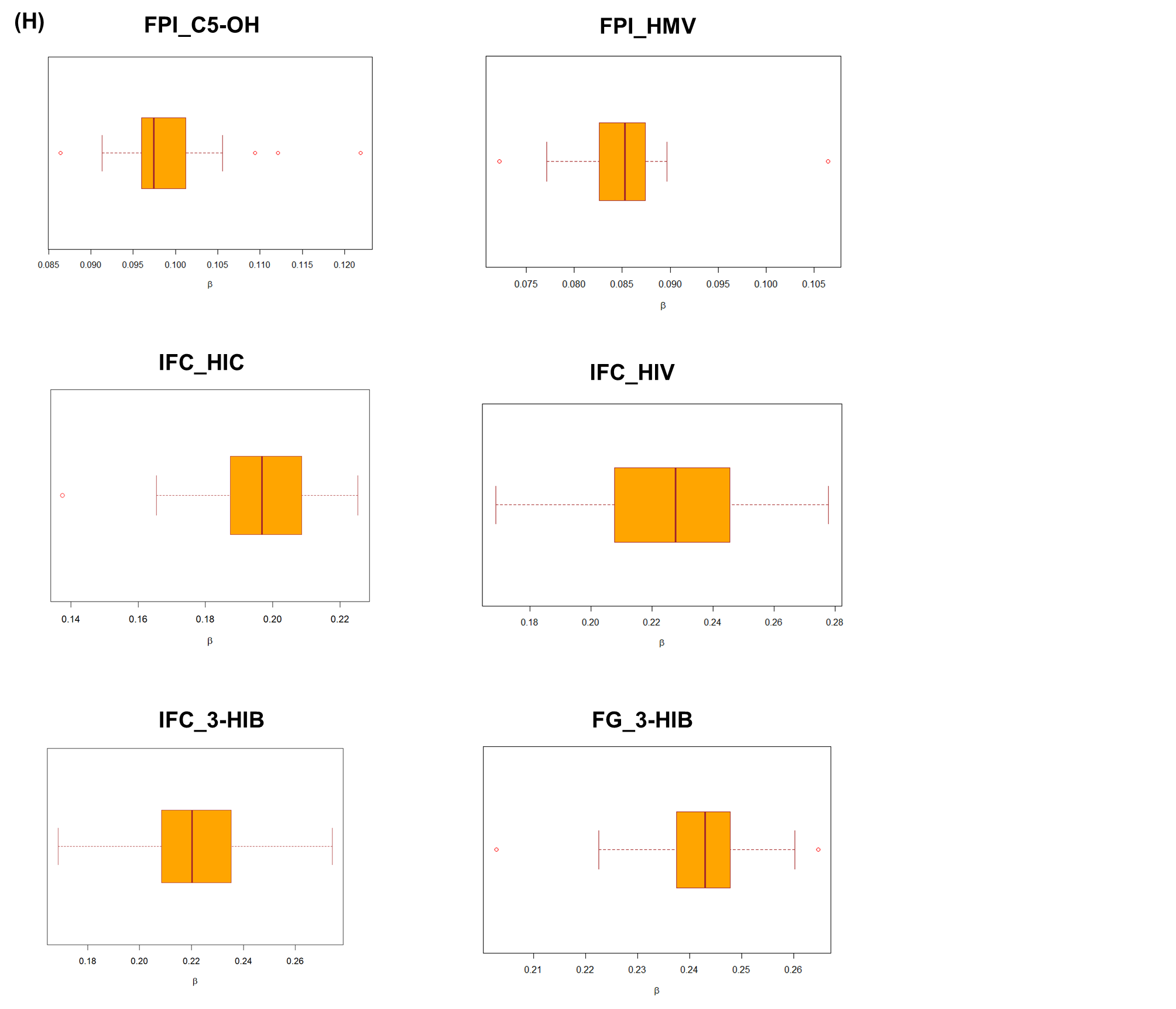
**

**Figure S6. Boxplots of leave-one-out analysis for statistically or suggestively significant association.** Adequately powered analysis of BCAAs in (A) forward and (B) reverse direction; Adequately powered analysis of BCAA catabolites in (C) forward and (D) reverse direction; Inadequate powered analysis of BCAAs in (E) forward and (F) reverse direction; Inadequate powered analysis of BCAA catabolites in (G) forward and (H) reverse direction. The box plots indicate the centralized trend of effect sizes (β) from the leave-one-out analysis, with the vertical line within the box representing the median, the left and right edges showing the first (Q1, the 25^th^ percentile) and third (Q3, the 75^th^ percentile) quartiles, respectively. The box width corresponds to the interquartile range (IQR = Q3 − Q1), and red points identify potentially outlying SNPs.

**Reference**

1. Willer CJ, Li Y, Abecasis GR. METAL: fast and efficient meta-analysis of genomewide association scans. *Bioinformatics.* 2010;**26**(17):2190-2191.

2. Bulik-Sullivan BK, Loh P-R, Finucane HK, et al. LD Score regression distinguishes confounding from polygenicity in genome-wide association studies. *Nat Genet.* 2015;**47**(3):291-295.

3. Yang J, Weedon MN, Purcell S, et al. Genomic inflation factors under polygenic inheritance. *Eur J Hum Gene*t*.* 2011;**19**(7):807-812.

4. Turley P, Walters RK, Maghzian O, et al. Multi-trait analysis of genome-wide association summary statistics using MTAG. *Nat Genet.* 2018;**50**(2):229-237.

5. cks2903. ValueError: The mean chi2 statistic of trait 1 is less than 1.02, which is too small to be well-suited for MTAG. Issue #39. JonJala/mtag. GitHub. Published August 17, 2018. Accessed January 21, 2026. https://github.com/JonJala/mtag/issues/39

6. Pierce BL, Ahsan H, VanderWeele TJ. Power and instrument strength requirements for Mendelian randomization studies using multiple genetic variants. *Int J Epidemio*l*.* 2011;**40**(3):740-752.

7. Hemani G, Tilling K, Davey Smith G. Orienting the causal relationship between imprecisely measured traits using GWAS summary data. *PLoS Genet.* 2017;**13**(11):e1007081.

8. Nguyen K, Mitchell BD. A guide to understanding mendelian randomization studies. *Arthritis Care Res.* 2024;**76**(11):1451.

9. Burgess S, Butterworth A, Thompson SG. Mendelian randomization analysis with multiple genetic variants using summarized data*. Genet Epidemiol.* 2013;**37**(7):658-665.

10. Greco M, Minelli C, Sheehan N, Thompson JR. Detecting pleiotropy in Mendelian randomisation studies with summary data and a continuous outcome. *Stat Med*. 2015; **34**(21), 2926-2940.

11. Bowden J, Davey Smith G, Haycock PC, Burgess S. Consistent estimation in Mendelian randomization with some invalid instruments using a weighted median estimator. *Genet Epidemiol.* 2016;**40**(4):304-314.

12. Burgess S, Foley CN, Allara E, Staley JR, Howson JM. A robust and efficient method for Mendelian randomization with hundreds of genetic variants. *Nat Commun.* 2020;**11**(1):376.

13. Bowden J, Davey Smith G, Burgess S. Mendelian randomization with invalid instruments: effect estimation and bias detection through Egger regression. *Int J Epidemiol.* 2015;**44**(2):512-525.

14. Verbanck M, Chen CY, Neale B, Do R. Detection of widespread horizontal pleiotropy in causal relationships inferred from Mendelian randomization between complex traits and diseases. *Nat Genet.* 2018;**50**(5):693-698.

15. Hemani G, Zheng J, Elsworth B, et al. The MR-Base platform supports systematic causal inference across the human phenome. *elife.* 2018;**7**:e34408.

16. Patel A, Ye T, Xue H, et al. MendelianRandomization v0. 9.0: updates to an R package for performing Mendelian randomization analyses using summarized data. *Wellcome Open Res.* 2023;**8**:449.

17. Burgess S. Sample size and power calculations in Mendelian randomization with a single instrumental variable and a binary outcome. *Int J Epidemiol.* 2014;**43**(3):922-929.

18. Giambartolomei C, Vukcevic D, Schadt EE, et al. Bayesian test for colocalisation between pairs of genetic association studies using summary statistics. *PLoS Genet*. 2014;**10**(5):e1004383.

19. Liebich HM, Först C. Hydroxycarboxylic and oxocarboxylic acids in urine: products from branched-chain amino acid degradation and from ketogenesis. *J Chromatogr B Biomed Sci Appl.* 1984;**309**:225-242.

20. Treacy E, Clow C, Reade T, Chitayat D, Mamer O, Scriver C. Maple syrup urine disease: interrelations between branched‐chain amino‐, oxo‐and hydroxyacids; implications for treatment; associations with CNS dysmyelination. *J Inherit Metab* *Dis.* 1992;**15**(1):121-135.

21. Heemskerk MM, Van Harmelen VJ, Van Dijk KW, Van Klinken JB. Reanalysis of mGWAS results and in vitro validation show that lactate dehydrogenase interacts with branched-chain amino acid metabolism. *Eur J Hum Genet.* 2016;**24**(1):142-145.

22. Mamer O, Reimer M. On the mechanisms of the formation of L-alloisoleucine and the 2-hydroxy-3-methylvaleric acid stereoisomers from L-isoleucine in maple syrup urine disease patients and in normal humans. *J Biol Chem.* 1992;**267**(31):22141-22147.
